# Correction: Association between socioeconomic background and cancer: An ecological study using cancer registry and various community socioeconomic status indicators in Kanagawa, Japan

**DOI:** 10.1371/journal.pone.0334930

**Published:** 2025-10-17

**Authors:** Satoru Kanda, Kaname Watanabe, Sho Nakamura, Hiroto Narimatsu

An additional affiliation is missing for the first author. Satoru Kanda is also affiliated with the Cancer Prevention and Control Division, Kanagawa Cancer Center Research Institute, Yokohama, Kanagawa, Japan. This is their primary affiliation.
