# Supplementary material for: Association between socioeconomic background and cancer: An ecological study using cancer registry and various community socioeconomic status indicators in Kanagawa, Japan
Source: PLoS One. 2025 Jul 9;20(7):e0326895. doi: 10.1371/journal.pone.0326895 (PMC12240336; doi:10.1371/journal.pone.0326895)
Supplement: S1 Data — S1 File. Community SES information. S1 Fig. Scatterplot of the relationship between community land price (A), neighborhood income (B), education level (C), and employment rate (D), with stomach cancer morbidity and mortality for men and women in Kanagawa, Japan, 2000–2015. Each plot shows data per year and community. 1$ = 133 Japanese Yen, the rate on March 20, 2023. S2 Fig. Scatterplot of the relationship between community land price (A), neighborhood income (B), education level (C), and employment rate (D), with colorectal cancer morbidity and mortality for men and women in Kanagawa, Japan, 2000–2015. Each plot shows data per year and community. 1$ = 133 Japanese Yen, the rate on March 20, 2023. S3 Fig. Scatterplot of the relationship between community land price (A), neighborhood income (B), education level (C), and employment rate (D), with liver cancer morbidity and mortality for men and women in Kanagawa, Japan, 2000–2015. Each plot shows data per year and community. 1$ = 133 Japanese Yen, the rate on March 20, 2023. S4 Fig. Scatterplot of the relationship between community land price (A), neighborhood income (B), education level (C), and employment rate (D), with breast cancer morbidity and mortality for women in Kanagawa, Japan, 2000–2015. Each plot shows data per year and community. 1$ = 133 Japanese Yen, the rate on March 20, 2023. S1 Table. Correlation coefficients of the aging rate, screening rate, and community SES indicators in Kanagawa, Japan, 2000–2015. S2 Table. VIF of the Poisson regression using community SES indicator, aging rate, and year as explanatory variables. S3 Table. VIF of the Poisson regression using community SES indicator, aging rate, year, and municipality code as explanatory variables. S4 Table. Multilevel analysis by the year for cancer morbidity in Kanagawa, Japan, 2000–2015. S5 Table. Multilevel analysis by the year for cancer mortality in Kanagawa, Japan, 2000–2015. S6 Table. Multilevel analysis by the municipality code for canc [file pone.0326895.s001.zip › S1_File.docx]

**S1 File. Community SES information.**

**Land price**

Land price is considered to reflect regional SES throughout a person's life. Land price was calculated as the price of land per square meter. We converted the land price to U.S. dollars (1$ = 133 Japanese Yen, the rate on March 20th, 2023). The land price data was taken from the following site: <http://www.pref.kanagawa.jp/docs/h4k/cnt/f4920/>. [Cited 2025 March 22].

| **city, town,** | **Land price, $×10^3^/m^2a^** | | | |
| --- | --- | --- | --- | --- |
|  | 2000 | 2005 | 2010 | 2015 |
| Tsurumi Ward, Yokohama City | 2.20 | 1.81 | 1.77 | 1.85 |
| Kanagawa Ward, Yokohama City | 2.29 | 1.74 | 1.70 | 1.73 |
| Nishi Ward, Yokohama City | 2.22 | 1.67 | 1.66 | 1.72 |
| Naka Ward, Yokohama City | 2.51 | 2.06 | 2.18 | 2.29 |
| Minami Ward, Yokohama City | 1.97 | 1.53 | 1.51 | 1.59 |
| Hodogaya Ward, Yokohama City | 1.77 | 1.41 | 1.40 | 1.42 |
| Isogo Ward, Yokohama City | 1.83 | 1.42 | 1.39 | 1.36 |
| Kanazawa Ward, Yokohama City | 1.77 | 1.43 | 1.40 | 1.39 |
| Kohoku Ward, Yokohama City | 2.35 | 2.08 | 2.15 | 2.26 |
| Totsuka Ward, Yokohama City | 1.65 | 1.31 | 1.28 | 1.28 |
| Konan Ward, Yokohama City | 1.83 | 1.45 | 1.43 | 1.42 |
| Asahi Ward, Yokohama City | 1.83 | 1.44 | 1.43 | 1.41 |
| Midori Ward, Yokohama City | 1.89 | 1.49 | 1.49 | 1.52 |
| Seya Ward, Yokohama City | 1.74 | 1.37 | 1.32 | 1.29 |
| Sakae Ward, Yokohama City | 1.66 | 1.30 | 1.32 | 1.32 |
| Izumi Ward, Yokohama City | 1.73 | 1.41 | 1.44 | 1.39 |
| Aoba Ward, Yokohama City | 2.24 | 1.94 | 2.07 | 2.10 |
| Tsuzuki Ward, Yokohama City | 1.95 | 1.60 | 1.70 | 1.72 |
| Kawasaki Ward, Kawasaki City | 2.49 | 2.03 | 2.03 | 2.02 |
| Saiwai Ward, Kawasaki City | 2.46 | 2.09 | 2.16 | 2.28 |
| Nakahara Ward, Kawasaki City | 2.42 | 2.09 | 2.39 | 2.60 |
| Takatsu Ward, Kawasaki City | 2.27 | 1.90 | 1.99 | 2.16 |
| Tama Ward, Kawasaki City | 2.04 | 1.52 | 1.58 | 1.66 |
| Miyamae Ward, Kawasaki City | 2.02 | 1.58 | 1.68 | 1.73 |
| Asao Ward, Kawasaki City | 1.83 | 1.38 | 1.38 | 1.31 |
| Midori Ward, Sagamihara City | NA | NA | 0.62 | 0.62 |
| Chuoku Ward, Sagamihara City | NA | NA | 1.03 | 1.03 |
| Minami Ward, Sagamihara City | NA | NA | 1.27 | 1.25 |
| Yokosuka City | 1.38 | 1.07 | 1.00 | 0.91 |
| Hiratsuka City | 1.47 | 1.17 | 1.11 | 0.97 |
| Kamakura City | 1.84 | 1.53 | 1.54 | 1.45 |
| Fuzisawa City | 1.82 | 1.51 | 1.53 | 1.53 |
| Odawara City | 1.26 | 1.03 | 0.95 | 0.82 |
| Chigasaki City | 1.78 | 1.48 | 1.52 | 1.41 |
| Zushi City | 1.86 | 1.36 | 1.30 | 1.29 |
| Miura City | 1.31 | 0.95 | 0.85 | 0.60 |
| Hadano City | 1.23 | 0.90 | 0.82 | 0.68 |
| Atsugi City | 1.32 | 1.00 | 0.96 | 0.84 |
| Yamato City | 1.73 | 1.35 | 1.33 | 1.26 |
| Isehara City | 1.33 | 0.97 | 0.92 | 0.81 |
| Ebina City | 1.53 | 1.17 | 1.11 | 1.04 |
| Zama City | 1.59 | 1.22 | 1.16 | 1.07 |
| Minamiashigara City | 1.07 | 0.89 | 0.78 | 0.50 |
| Ayase City | 1.42 | 1.07 | 1.01 | 0.87 |
| Hayama Town | 1.50 | 1.20 | 1.17 | 1.04 |
| Samukawa Town | 1.39 | 1.08 | 1.05 | 0.88 |
| Oiso Town | 1.47 | 1.22 | 1.17 | 0.92 |
| Ninomiya Town | 1.24 | 0.94 | 0.87 | 0.70 |
| Nakai Town | 0.97 | 0.66 | 0.56 | 0.39 |
| Oi Town | 0.95 | 0.80 | 0.72 | 0.54 |
| Matsuda Town | 1.12 | 0.91 | 0.85 | 0.78 |
| Yamakita Town | 0.76 | 0.55 | 0.46 | 0.38 |
| Kaise Town | 1.12 | 0.95 | 0.88 | 0.66 |
| Hakone Town | 0.50 | 0.35 | 0.33 | 0.31 |
| Manazuru Town | 0.94 | 0.71 | 0.64 | 0.50 |
| Yugawara Town | 1.02 | 0.76 | 0.69 | 0.59 |
| Aikawa Town | 0.84 | 0.56 | 0.53 | 0.41 |
| Kiyokawa Village | 0.59 | 0.32 | 0.27 | 0.25 |

a Land price by year and community for each year, 1$ = 133 Japanese Yen, the rate on March 20th, 2023)

**Neighborhood income**

Neighborhood income is considered to reflect regional SES after graduation. The obtained income data were the number of families with incomes of less than 3 000 000, 3 000 000-5 000 000, 5 000 000-7 000 000, 7 000 000-10 000 000, 10 000 000-15 000 000, and more than 15 000 000 Japanese Yen per community. We defined each income as 1 500 000, 4 000 000, 6 000 000, 8 500 000, 12 500 000, and 20 000 000 Japanese Yen, respectively, and calculated the average income by multiplying each income and number of families per income, summing them up, and finally dividing by the number of all families in each year and community. We defined those average incomes as the neighborhood income. For example, the average income in a given region and year was calculated as follows:

$$\text{Neighborhood income}$$

$$= (1 500 000 \times number of families with income less than 3 000 000 Japanese Yen$$

$$+ 4 000 000 \times number of families with family income form 3 000 000 to 5 000 000$$

$$+ 6 000 000 \times number of families with family income form 5 000 000 to 7 000 000$$

$$+ 8 500 000 \times number of families with family income form 7 000 000 to 10 000 000$$

$$+ 12 500 000 \times number of families with family income form 10 000 000 to 15 000 000$$

$$+ 20 000 000 \times number of families with income more than 15 000 000)$$

$$/ the number of all families$$

Since income data was only available every five years starting in 2003, the analysis was conducted by matching income data and cancer data at three-year intervals, for example, matching income data in 2003 with cancer data in 2000. We converted neighborhood income to U.S. dollars (1$ = 133 Japanese Yen, the rate on March 20^th^, 2023). The income data was taken from the following site: https://www.e-stat.go.jp/stat-search/database?page=1&toukei=00200522&tstat=000001127155. [Cited 2025 March 22].

| **city, town,** | **Neighborhood income, $×10^3b^** | | | |
| --- | --- | --- | --- | --- |
|  | 2000 | 2005 | 2010 | 2015 |
| Tsurumi Ward, Yokohama City | 40.2 | 41.7 | 38.8 | 40.3 |
| Kanagawa Ward, Yokohama City | 41.3 | 42.4 | 37.8 | 39.1 |
| Nishi Ward, Yokohama City | 38.8 | 41.0 | 41.1 | 42.8 |
| Naka Ward, Yokohama City | 42.4 | 46.0 | 41.4 | 41.3 |
| Minami Ward, Yokohama City | 39.7 | 39.6 | 34.2 | 34.6 |
| Hodogaya Ward, Yokohama City | 42.1 | 40.9 | 37.2 | 39.5 |
| Isogo Ward, Yokohama City | 44.0 | 42.0 | 38.2 | 38.8 |
| Kanazawa Ward, Yokohama City | 47.0 | 46.0 | 41.5 | 41.4 |
| Kohoku Ward, Yokohama City | 45.2 | 45.6 | 42.5 | 44.2 |
| Totsuka Ward, Yokohama City | 44.7 | 44.9 | 41.6 | 42.5 |
| Konan Ward, Yokohama City | 47.8 | 45.6 | 40.3 | 40.2 |
| Asahi Ward, Yokohama City | 44.1 | 43.5 | 38.5 | 38.7 |
| Midori Ward, Yokohama City | 44.8 | 43.5 | 39.8 | 41.1 |
| Seya Ward, Yokohama City | 41.3 | 39.2 | 36.1 | 36.4 |
| Sakae Ward, Yokohama City | 46.6 | 45.8 | 40.3 | 40.0 |
| Izumi Ward, Yokohama City | 47.7 | 46.3 | 40.2 | 42.1 |
| Aoba Ward, Yokohama City | 55.4 | 56.6 | 52.2 | 50.3 |
| Tsuzuki Ward, Yokohama City | 54.0 | 54.7 | 51.1 | 53.4 |
| Kawasaki Ward, Kawasaki City | 37.4 | 36.1 | 33.4 | 35.6 |
| Saiwai Ward, Kawasaki City | 39.7 | 45.9 | 38.5 | 41.7 |
| Nakahara Ward, Kawasaki City | 41.6 | 45.8 | 44.1 | 45.7 |
| Takatsu Ward, Kawasaki City | 42.8 | 45.8 | 39.1 | 41.5 |
| Tama Ward, Kawasaki City | 39.3 | 42.1 | 36.6 | 38.4 |
| Miyamae Ward, Kawasaki City | 49.1 | 46.5 | 42.1 | 45.5 |
| Asao Ward, Kawasaki City | 49.4 | 49.7 | 45.2 | 46.0 |
| Midori Ward, Sagamihara City | NA | NA | 36.0 | 36.3 |
| Chuoku Ward, Sagamihara City | NA | NA | 34.9 | 35.8 |
| Minami Ward, Sagamihara City | NA | NA | 36.8 | 37.5 |
| Yokosuka City | 41.7 | 39.2 | 34.0 | 35.1 |
| Hiratsuka City | 41.7 | 40.2 | 35.3 | 36.5 |
| Kamakura City | 48.3 | 47.9 | 43.8 | 45.3 |
| Fuzisawa City | 43.8 | 43.4 | 39.7 | 40.7 |
| Odawara City | 43.8 | 41.1 | 35.7 | 36.7 |
| Chigasaki City | 44.4 | 42.9 | 39.2 | 41.4 |
| Zushi City | 47.8 | 47.1 | 42.2 | 43.1 |
| Miura City | 40.7 | 39.4 | 33.4 | 33.5 |
| Hadano City | 40.8 | 38.0 | 34.8 | 35.2 |
| Atsugi City | 41.9 | 39.5 | 35.7 | 37.2 |
| Yamato city | 40.1 | 39.4 | 36.1 | 37.0 |
| Isehara City | 41.7 | 40.3 | 35.8 | 37.9 |
| Ebina City | 47.8 | 44.7 | 37.9 | 40.0 |
| Zama City | 42.2 | 41.7 | 34.8 | 34.6 |
| Minamiashigara City | 46.9 | 44.4 | 38.4 | 43.0 |
| Ayase City | 43.2 | 40.0 | 35.7 | 36.0 |
| Hayama Town | 51.6 | 45.7 | 44.1 | 42.5 |
| Samukawa Town | 43.2 | 40.6 | 35.4 | 36.1 |
| Oiso Town | 46.5 | 45.0 | 39.6 | 41.4 |
| Ninomiya Town | 47.2 | 45.4 | 38.7 | 38.4 |
| Nakai Town | NA | NA | NA | NA |
| Oi Town | 46.1 | 43.8 | 35.5 | 40.8 |
| Matsuda Town | NA | NA | NA | NA |
| Yamakita Town | NA | NA | NA | NA |
| Kaise Town | NA | 41.6 | 37.7 | 42.2 |
| Hakone Town | 35.0 | NA | NA | NA |
| Manazuru Town | NA | NA | NA | NA |
| Yugawara Town | 35.9 | 33.1 | 30.4 | 28.2 |
| Aikawa Town | 41.9 | 37.5 | 31.4 | 33.6 |
| Kiyokawa Village | NA | NA | NA | NA |

b Neighborhood income by year and community for each year, 1$ = 133 Japanese Yen, the rate on March 20th, 2023)

**Education level**

Education level is considered to reflect regional SES in childhood. We defined the community education level as the proportion of people with more than a high school education in each community. In some areas of Kanagawa Prefecture during the period covered by the data used in this study, the percentage of college graduates in the population was low at about 15%, and it was possible that those who went on to college alone would not cover the high educational level of the population at that time. Therefore, in this study, we defined higher education level as those who were educated in higher educational institutions than high school. The population of people with more than a high school education was taken from the following site: <https://www.e-stat.go.jp/stat-search?page=1&toukei=00200521>. [Cited 2025 March 22]. Education levels were calculated by dividing that data by the total population.

| **city, town,** | **Education level, %^c^** | | | |
| --- | --- | --- | --- | --- |
|  | 2000 | 2005 | 2010 | 2015 |
| Tsurumi Ward, Yokohama City | 33.5 | NA | 36.3 | NA |
| Kanagawa Ward, Yokohama City | 38.6 | NA | 42.5 | NA |
| Nishi Ward, Yokohama City | 36.4 | NA | 43.7 | NA |
| Naka Ward, Yokohama City | 34.3 | NA | 37.7 | NA |
| Minami Ward, Yokohama City | 32.8 | NA | 35.0 | NA |
| Hodogaya Ward, Yokohama City | 37.4 | NA | 39.3 | NA |
| Isogo Ward, Yokohama City | 38.7 | NA | 39.0 | NA |
| Kanazawa Ward, Yokohama City | 44.0 | NA | 45.6 | NA |
| Kohoku Ward, Yokohama City | 48.8 | NA | 50.3 | NA |
| Totsuka Ward, Yokohama City | 41.3 | NA | 45.2 | NA |
| Konan Ward, Yokohama City | 40.4 | NA | 42.7 | NA |
| Asahi Ward, Yokohama City | 37.7 | NA | 38.8 | NA |
| Midori Ward, Yokohama City | 40.4 | NA | 42.5 | NA |
| Seya Ward, Yokohama City | 32.0 | NA | 33.7 | NA |
| Sakae Ward, Yokohama City | 43.2 | NA | 45.8 | NA |
| Izumi Ward, Yokohama City | 36.5 | NA | 40.0 | NA |
| Aoba Ward, Yokohama City | 58.8 | NA | 58.4 | NA |
| Tsuzuki Ward, Yokohama City | 48.5 | NA | 51.3 | NA |
| Kawasaki Ward, Kawasaki City | 23.7 | NA | 25.4 | NA |
| Saiwai Ward, Kawasaki City | 29.6 | NA | 34.7 | NA |
| Nakahara Ward, Kawasaki City | 43.0 | NA | 44.9 | NA |
| Takatsu Ward, Kawasaki City | 41.6 | NA | 40.5 | NA |
| Tama Ward, Kawasaki City | 48.1 | NA | 42.6 | NA |
| Miyamae Ward, Kawasaki City | 48.2 | NA | 43.8 | NA |
| Asao Ward, Kawasaki City | 53.1 | NA | 51.5 | NA |
| Midori Ward, Sagamihara City | NA | NA | 31.7 | NA |
| Chuoku Ward, Sagamihara City | NA | NA | 31.8 | NA |
| Minami Ward, Sagamihara City | NA | NA | 40.6 | NA |
| Yokosuka City | 27.6 | NA | 28.9 | NA |
| Hiratsuka City | 30.5 | NA | 33.9 | NA |
| Kamakura City | 50.6 | NA | 51.2 | NA |
| Fuzisawa City | 41.1 | NA | 44.0 | NA |
| Odawara City | 29.4 | NA | 33.4 | NA |
| Chigasaki City | 40.4 | NA | 42.5 | NA |
| Zushi City | 48.2 | NA | 50.2 | NA |
| Miura City | 20.1 | NA | 23.7 | NA |
| Hadano City | 31.0 | NA | 34.3 | NA |
| Atsugi City | 34.6 | NA | 36.1 | NA |
| Yamato city | 34.8 | NA | 35.9 | NA |
| Isehara City | 37.7 | NA | 40.4 | NA |
| Ebina City | 36.6 | NA | 39.4 | NA |
| Zama City | 35.4 | NA | 34.9 | NA |
| Minamiashigara City | 26.9 | NA | 30.9 | NA |
| Ayase City | 26.4 | NA | 29.1 | NA |
| Hayama Town | 45.8 | NA | 52.6 | NA |
| Samukawa Town | 25.9 | NA | 28.2 | NA |
| Oiso Town | 39.8 | NA | 44.7 | NA |
| Ninomiya Town | 39.1 | NA | 42.8 | NA |
| Nakai Town | 26.4 | NA | 28.5 | NA |
| Oi Town | 30.8 | NA | 31.4 | NA |
| Matsuda Town | 28.5 | NA | 30.4 | NA |
| Yamakita Town | 22.7 | NA | 27.4 | NA |
| Kaise Town | 28.0 | NA | 36.1 | NA |
| Hakone Town | 20.1 | NA | 26.1 | NA |
| Manazuru Town | 23.6 | NA | 28.1 | NA |
| Yugawara Town | 23.0 | NA | 27.5 | NA |
| Aikawa Town | 20.2 | NA | 22.2 | NA |
| Kiyokawa Village | 21.5 | NA | 25.1 | NA |

c Education level by year and community for each year **Employment rate**

Employment rate is considered to reflect the regional SES of the working-age generation. The employment rate is defined as the number of people in jobs in a community divided by the population aged 15 and over, excluding students and those engaged in domestic work, in that community. In community SES data, only employment rates can use gender-disaggregated data. The employment rate data was calculated from the data in the following site: <https://www.e-stat.go.jp/stat-search?page=1&toukei=00200521>. [Cited 2025 March 22].

| **city, town,** | **Employment rate (Men), %^d^** | | | |
| --- | --- | --- | --- | --- |
|  | 2000 | 2005 | 2010 | 2015 |
| Tsurumi Ward, Yokohama City | 95.5 | 94.2 | 94.0 | 96.2 |
| Kanagawa Ward, Yokohama City | 95.4 | 94.1 | 94.1 | 96.0 |
| Nishi Ward, Yokohama City | 94.7 | 94.2 | 93.7 | 96.1 |
| Naka Ward, Yokohama City | 89.8 | 89.8 | 92.1 | 95.0 |
| Minami Ward, Yokohama City | 93.4 | 92.4 | 92.6 | 94.8 |
| Hodogaya Ward, Yokohama City | 95.4 | 94.5 | 93.4 | 95.7 |
| Isogo Ward, Yokohama City | 95.2 | 94.6 | 93.5 | 95.4 |
| Kanazawa Ward, Yokohama City | 94.9 | 94.8 | 94.1 | 95.4 |
| Kohoku Ward, Yokohama City | 96.3 | 95.2 | 94.7 | 96.5 |
| Totsuka Ward, Yokohama City | 95.6 | 94.9 | 94.3 | 96.0 |
| Konan Ward, Yokohama City | 95.1 | 93.9 | 93.6 | 95.6 |
| Asahi Ward, Yokohama City | 95.1 | 94.2 | 92.8 | 95.5 |
| Midori Ward, Yokohama City | 95.8 | 94.8 | 93.8 | 95.9 |
| Seya Ward, Yokohama City | 94.2 | 92.3 | 92.7 | 95.2 |
| Sakae Ward, Yokohama City | 94.8 | 94.2 | 93.7 | 95.3 |
| Izumi Ward, Yokohama City | 95.0 | 94.0 | 93.5 | 95.2 |
| Aoba Ward, Yokohama City | 96.6 | 96.2 | 95.3 | 96.7 |
| Tsuzuki Ward, Yokohama City | 96.8 | 96.2 | 95.9 | 97.2 |
| Kawasaki Ward, Kawasaki City | 93.0 | 92.0 | 92.8 | 93.8 |
| Saiwai Ward, Kawasaki City | 93.4 | 90.8 | 93.9 | 95.7 |
| Nakahara Ward, Kawasaki City | 96.1 | 95.4 | 95.5 | 97.1 |
| Takatsu Ward, Kawasaki City | 95.0 | 94.8 | 94.5 | 96.4 |
| Tama Ward, Kawasaki City | 95.2 | 94.9 | 94.2 | 95.9 |
| Miyamae Ward, Kawasaki City | 95.3 | 95.2 | 94.9 | 96.5 |
| Asao Ward, Kawasaki City | 95.8 | 95.1 | 94.7 | 96.4 |
| Midori Ward, Sagamihara City | NA | NA | 93.8 | 95.4 |
| Chuoku Ward, Sagamihara City | NA | NA | 93.3 | 94.6 |
| Minami Ward, Sagamihara City | NA | NA | 93.6 | 95.2 |
| Yokosuka City | 94.0 | 93.2 | 92.3 | 94.5 |
| Hiratsuka City | 93.1 | 93.0 | 91.9 | 95.2 |
| Kamakura City | 95.6 | 94.8 | 94.5 | 96.2 |
| Fuzisawa City | 95.6 | 94.2 | 94.1 | 96.0 |
| Odawara City | 95.2 | 94.3 | 93.0 | 95.4 |
| Chigasaki City | 94.8 | 94.5 | 93.9 | 95.6 |
| Zushi City | 95.3 | 93.8 | 94.1 | 95.8 |
| Miura City | 94.3 | 93.7 | 91.9 | 94.2 |
| Hadano City | 95.7 | 94.0 | 91.6 | 94.1 |
| Atsugi City | 94.9 | 93.9 | 92.0 | 95.2 |
| Yamato city | 94.7 | 93.5 | 93.1 | 95.3 |
| Isehara City | 94.0 | 93.2 | 91.5 | 95.7 |
| Ebina City | 95.2 | 93.9 | 93.5 | 95.7 |
| Zama City | 95.1 | 94.5 | 93.3 | 95.0 |
| Minamiashigara City | 95.8 | 94.4 | 91.9 | 95.1 |
| Ayase City | 94.7 | 93.6 | 92.6 | 94.9 |
| Hayama Town | 95.3 | 94.1 | 92.7 | 96.3 |
| Samukawa Town | 95.0 | 93.4 | 93.6 | 95.2 |
| Oiso Town | 95.0 | 93.9 | 93.1 | 95.0 |
| Ninomiya Town | 95.3 | 94.0 | 92.9 | 95.2 |
| Nakai Town | 96.6 | 95.6 | 94.5 | 95.7 |
| Oi Town | 96.3 | 95.4 | 93.0 | 94.9 |
| Matsuda Town | 95.1 | 94.3 | 93.1 | 94.5 |
| Yamakita Town | 95.8 | 94.1 | 93.0 | 94.7 |
| Kaise Town | 95.7 | 94.3 | 93.5 | 95.6 |
| Hakone Town | 96.6 | 95.8 | 95.2 | 96.7 |
| Manazuru Town | 93.8 | 92.0 | 91.4 | 93.7 |
| Yugawara Town | 93.8 | 92.9 | 90.0 | 93.5 |
| Aikawa Town | 94.2 | 93.1 | 90.3 | 94.3 |
| Kiyokawa Village | 96.3 | 95.2 | 94.3 | 95.5 |

d Employment rate in men by year and community for each year

| **city, town,** | **Employment rate (Women), %^e^** | | | |
| --- | --- | --- | --- | --- |
|  | 2000 | 2005 | 2010 | 2015 |
| Tsurumi Ward, Yokohama City | 95.7 | 95.0 | 95.3 | 97.0 |
| Kanagawa Ward, Yokohama City | 95.8 | 95.2 | 95.4 | 96.9 |
| Nishi Ward, Yokohama City | 95.3 | 95.0 | 95.1 | 96.7 |
| Naka Ward, Yokohama City | 94.7 | 93.7 | 94.6 | 96.1 |
| Minami Ward, Yokohama City | 94.7 | 94.3 | 94.4 | 96.2 |
| Hodogaya Ward, Yokohama City | 95.7 | 95.4 | 95.4 | 96.6 |
| Isogo Ward, Yokohama City | 95.4 | 95.4 | 94.8 | 96.5 |
| Kanazawa Ward, Yokohama City | 95.8 | 95.7 | 95.8 | 97.1 |
| Kohoku Ward, Yokohama City | 95.8 | 95.6 | 95.6 | 97.1 |
| Totsuka Ward, Yokohama City | 95.9 | 95.4 | 95.5 | 96.9 |
| Konan Ward, Yokohama City | 95.4 | 95.2 | 95.0 | 96.8 |
| Asahi Ward, Yokohama City | 95.6 | 95.3 | 95.0 | 96.5 |
| Midori Ward, Yokohama City | 95.6 | 95.3 | 95.2 | 96.9 |
| Seya Ward, Yokohama City | 94.6 | 93.8 | 94.5 | 96.2 |
| Sakae Ward, Yokohama City | 95.9 | 95.1 | 95.1 | 96.7 |
| Izumi Ward, Yokohama City | 95.6 | 95.1 | 95.2 | 96.6 |
| Aoba Ward, Yokohama City | 95.9 | 96.1 | 95.8 | 97.3 |
| Tsuzuki Ward, Yokohama City | 96.0 | 96.0 | 96.1 | 97.4 |
| Kawasaki Ward, Kawasaki City | 94.7 | 94.1 | 94.8 | 95.7 |
| Saiwai Ward, Kawasaki City | 94.9 | 93.6 | 95.2 | 96.9 |
| Nakahara Ward, Kawasaki City | 95.6 | 95.9 | 95.8 | 97.3 |
| Takatsu Ward, Kawasaki City | 95.0 | 95.4 | 95.6 | 96.9 |
| Tama Ward, Kawasaki City | 95.5 | 95.8 | 95.5 | 96.9 |
| Miyamae Ward, Kawasaki City | 95.2 | 95.6 | 95.5 | 97.1 |
| Asao Ward, Kawasaki City | 95.7 | 95.7 | 95.5 | 97.0 |
| Midori Ward, Sagamihara City | NA | NA | 95.8 | 96.8 |
| Chuoku Ward, Sagamihara City | NA | NA | 95.3 | 96.3 |
| Minami Ward, Sagamihara City | NA | NA | 95.2 | 96.6 |
| Yokosuka City | 95.2 | 94.8 | 94.4 | 96.1 |
| Hiratsuka City | 94.9 | 95.0 | 94.5 | 96.6 |
| Kamakura City | 96.0 | 95.8 | 95.7 | 97.0 |
| Fuzisawa City | 95.9 | 95.3 | 95.3 | 96.9 |
| Odawara City | 96.5 | 96.0 | 95.8 | 97.0 |
| Chigasaki City | 95.6 | 95.4 | 95.5 | 96.8 |
| Zushi City | 95.9 | 95.3 | 95.9 | 97.0 |
| Miura City | 96.2 | 96.0 | 95.0 | 96.5 |
| Hadano City | 96.6 | 95.4 | 94.8 | 96.3 |
| Atsugi City | 95.6 | 95.2 | 94.5 | 96.6 |
| Yamato city | 95.1 | 94.3 | 94.8 | 96.2 |
| Isehara City | 95.7 | 95.5 | 95.0 | 96.9 |
| Ebina City | 95.8 | 94.9 | 95.2 | 96.7 |
| Zama City | 95.6 | 95.3 | 94.9 | 96.3 |
| Minamiashigara City | 96.8 | 96.2 | 95.4 | 96.7 |
| Ayase City | 95.3 | 94.7 | 94.4 | 96.2 |
| Hayama Town | 96.4 | 95.9 | 94.7 | 97.1 |
| Samukawa Town | 96.2 | 94.9 | 95.5 | 97.0 |
| Oiso Town | 96.7 | 96.2 | 95.8 | 97.2 |
| Ninomiya Town | 96.3 | 96.0 | 95.9 | 96.8 |
| Nakai Town | 98.0 | 97.4 | 96.7 | 97.2 |
| Oi Town | 96.8 | 96.1 | 95.5 | 96.9 |
| Matsuda Town | 96.8 | 95.8 | 95.5 | 96.5 |
| Yamakita Town | 97.6 | 96.9 | 96.8 | 97.2 |
| Kaise Town | 96.1 | 95.7 | 95.1 | 96.6 |
| Hakone Town | 98.0 | 97.2 | 97.5 | 98.4 |
| Manazuru Town | 96.6 | 96.4 | 95.4 | 96.5 |
| Yugawara Town | 96.0 | 95.3 | 94.5 | 96.5 |
| Aikawa Town | 96.1 | 94.8 | 94.5 | 96.5 |
| Kiyokawa Village | 98.1 | 96.1 | 97.2 | 98.1 |

e Employment rate in women by year and community for each year
