# Supplementary material for: Association between socioeconomic background and cancer: An ecological study using cancer registry and various community socioeconomic status indicators in Kanagawa, Japan
Source: PLoS One. 2025 Jul 9;20(7):e0326895. doi: 10.1371/journal.pone.0326895 (PMC12240336; doi:10.1371/journal.pone.0326895)
Supplement: S1 Data — S1 File. Community SES information. S1 Fig. Scatterplot of the relationship between community land price (A), neighborhood income (B), education level (C), and employment rate (D), with stomach cancer morbidity and mortality for men and women in Kanagawa, Japan, 2000–2015. Each plot shows data per year and community. 1$ = 133 Japanese Yen, the rate on March 20, 2023. S2 Fig. Scatterplot of the relationship between community land price (A), neighborhood income (B), education level (C), and employment rate (D), with colorectal cancer morbidity and mortality for men and women in Kanagawa, Japan, 2000–2015. Each plot shows data per year and community. 1$ = 133 Japanese Yen, the rate on March 20, 2023. S3 Fig. Scatterplot of the relationship between community land price (A), neighborhood income (B), education level (C), and employment rate (D), with liver cancer morbidity and mortality for men and women in Kanagawa, Japan, 2000–2015. Each plot shows data per year and community. 1$ = 133 Japanese Yen, the rate on March 20, 2023. S4 Fig. Scatterplot of the relationship between community land price (A), neighborhood income (B), education level (C), and employment rate (D), with breast cancer morbidity and mortality for women in Kanagawa, Japan, 2000–2015. Each plot shows data per year and community. 1$ = 133 Japanese Yen, the rate on March 20, 2023. S1 Table. Correlation coefficients of the aging rate, screening rate, and community SES indicators in Kanagawa, Japan, 2000–2015. S2 Table. VIF of the Poisson regression using community SES indicator, aging rate, and year as explanatory variables. S3 Table. VIF of the Poisson regression using community SES indicator, aging rate, year, and municipality code as explanatory variables. S4 Table. Multilevel analysis by the year for cancer morbidity in Kanagawa, Japan, 2000–2015. S5 Table. Multilevel analysis by the year for cancer mortality in Kanagawa, Japan, 2000–2015. S6 Table. Multilevel analysis by the municipality code for canc [file pone.0326895.s001.zip › S1_Table.docx]

**S1 Table.** **Correlation coefficients of the aging rate, screening rate, and community SES indicators in Kanagawa, Japan, 2000-2015.**

| **Characteristics** | | | Aging rate  (95% CI) | Screening rate  (95% CI) | Land price  (95% CI) | Neighborhood  Income  (95% CI) | Education level  (95% CI) | Employment rate in men  (95% CI) | Employment rate in women  (95% CI) |
| --- | --- | --- | --- | --- | --- | --- | --- | --- | --- |
| Aging rate | | | 1.00 |  |  |  |  |  |  |
| Screening rate | | | -0.08  (-0.48, 0.35) | 1.00 |  |  |  |  |  |
| Land price | | | -0.49  (-0.62, -0.32) | 0.06  (-0.37, 0.47) | 1.00 |  |  |  |  |
| Neighborhood income | | | -0.53  (-0.66, -0.37) | 0.03  (-0.39, 0.45) | 0.47  (0.30, 0.61) | 1.00 |  |  |  |
| Education level | | | -0.07  (-0.26, 0.12) | -0.07  (-0.48, 0.36) | 0.41  (0.24, 0.56) | 0.59  (0.44, 0.70) | 1.00 |  |  |
| Employment rate in men | | | -0.65  (-0.75, -0.52) | 0.02  (-0.25, 0.29) | 0.40  (0.22, 0.56) | 0.72  (0.61, 0.80) | 0.37  (0.18, 0.53) | 1.00 |  |
| Employment rate in women | | | -0.29  (-0.47, -0.10) | -0.02  (-0.29, 0.25) | -0.06  (-0.25, 0.14) | 0.50  (0.34, 0.64) | 0.09  (-0.11, 0.29) | 0.72  (0.61, 0.81) | 1.00 |
|  |  |  |  |  |  |  |  |  |  |

SES indicates Socioeconomic status; CI, confidence interval.
