# Supplementary material for: Association between socioeconomic background and cancer: An ecological study using cancer registry and various community socioeconomic status indicators in Kanagawa, Japan
Source: PLoS One. 2025 Jul 9;20(7):e0326895. doi: 10.1371/journal.pone.0326895 (PMC12240336; doi:10.1371/journal.pone.0326895)
Supplement: S1 Data — S1 File. Community SES information. S1 Fig. Scatterplot of the relationship between community land price (A), neighborhood income (B), education level (C), and employment rate (D), with stomach cancer morbidity and mortality for men and women in Kanagawa, Japan, 2000–2015. Each plot shows data per year and community. 1$ = 133 Japanese Yen, the rate on March 20, 2023. S2 Fig. Scatterplot of the relationship between community land price (A), neighborhood income (B), education level (C), and employment rate (D), with colorectal cancer morbidity and mortality for men and women in Kanagawa, Japan, 2000–2015. Each plot shows data per year and community. 1$ = 133 Japanese Yen, the rate on March 20, 2023. S3 Fig. Scatterplot of the relationship between community land price (A), neighborhood income (B), education level (C), and employment rate (D), with liver cancer morbidity and mortality for men and women in Kanagawa, Japan, 2000–2015. Each plot shows data per year and community. 1$ = 133 Japanese Yen, the rate on March 20, 2023. S4 Fig. Scatterplot of the relationship between community land price (A), neighborhood income (B), education level (C), and employment rate (D), with breast cancer morbidity and mortality for women in Kanagawa, Japan, 2000–2015. Each plot shows data per year and community. 1$ = 133 Japanese Yen, the rate on March 20, 2023. S1 Table. Correlation coefficients of the aging rate, screening rate, and community SES indicators in Kanagawa, Japan, 2000–2015. S2 Table. VIF of the Poisson regression using community SES indicator, aging rate, and year as explanatory variables. S3 Table. VIF of the Poisson regression using community SES indicator, aging rate, year, and municipality code as explanatory variables. S4 Table. Multilevel analysis by the year for cancer morbidity in Kanagawa, Japan, 2000–2015. S5 Table. Multilevel analysis by the year for cancer mortality in Kanagawa, Japan, 2000–2015. S6 Table. Multilevel analysis by the municipality code for canc [file pone.0326895.s001.zip › S10_Table.docx]

**S10 Table. Linear regression with SES quartiles, year, and aging rate as explanatory variables and cancer incidence or mortality as response variables.**

| Morbidity | Sex | SES  quantile^a^ | Land price model^b^ | |  | Neighborhood income model^c^ | |
| --- | --- | --- | --- | --- | --- | --- | --- |
|  |  |  | β^d^ | 95% CI |  | β | 95% CI |
| Lung cancer |  |  |  |  |  |  |  |
|  | Men |  |  |  |  |  |  |
|  |  | quantile 1 | (reference) |  |  | (reference) |  |
|  |  | quantile 2 | 0.32 | -4.51, 5.15 |  | 3.77 | 0.58, 6.97 |
|  |  | quantile 3 | -0.96 | -5.78, 3.85 |  | 0.83 | -2.60, 4.26 |
|  |  | quantile 4 | -5.88 | -11.77, 0.01 |  | -4.41 | -8.01, -0.81 |
|  | Women |  |  |  |  |  |  |
|  |  | quantile 1 | (reference) |  |  | (reference) |  |
|  |  | quantile 2 | 4.17 | 1.57, 6.76 |  | 2.02 | 0.12, 3.92 |
|  |  | quantile 3 | 3.99 | 1.40, 6.58 |  | 1.11 | -0.94, 3.16 |
|  |  | quantile 4 | 1.79 | -1.28, 4.85 |  | -0.46 | -2.64, 1.72 |
| Stomach cancer |  |  |  |  |  |  |  |
|  | Men |  |  |  |  |  |  |
|  |  | quantile 1 | (reference) |  |  | (reference) |  |
|  |  | quantile 2 | -1.15 | -7.33, 5.03 |  | 3.05 | -1.61, 7.70 |
|  |  | quantile 3 | 1.38 | -4.78, 7.53 |  | 0.94 | -4.05, 5.94 |
|  |  | quantile 4 | -14.44 | -21.97, -6.91 |  | -7.28 | -12.52, -2.03 |
|  | Women |  |  |  |  |  |  |
|  |  | quantile 1 | (reference) |  |  | (reference) |  |
|  |  | quantile 2 | 0.36 | -3.26, 3.98 |  | 0.99 | -1.32, 3.30 |
|  |  | quantile 3 | 0.42 | -3.20, 4.03 |  | -0.46 | -2.95, 2.03 |
|  |  | quantile 4 | -5.04 | -9.31, -0.76 |  | -3.50 | -6.15, -0.85 |
| Colorectal cancer |  |  |  |  |  |  |  |
|  | Men |  |  |  |  |  |  |
|  |  | quantile 1 | (reference) |  |  | (reference) |  |
|  |  | quantile 2 | 14.31 | 7.12, 21.51 |  | 6.24 | 0.69, 11.78 |
|  |  | quantile 3 | 17.50 | 10.33, 24.67 |  | 1.78 | -7.73, 4.18 |
|  |  | quantile 4 | 10.81 | 2.04, 19.57 |  | -9.72 | -15.97, -3.46 |
|  | Women |  |  |  |  |  |  |
|  |  | quantile 1 | (reference) |  |  | (reference) |  |
|  |  | quantile 2 | -0.09 | -5.05, 4.86 |  | 4.16 | 0.38, 7.94 |
|  |  | quantile 3 | 5.41 | 0.47, 10.36 |  | 0.04 | -4.03, 4.12 |
|  |  | quantile 4 | -1.58 | -7.43, 4.27 |  | -5.23 | -9.56, -0.90 |
| Liver cancer |  |  |  |  |  |  |  |
|  | Men |  |  |  |  |  |  |
|  |  | quantile 1 | (reference) |  |  | (reference) |  |
|  |  | quantile 2 | -0.10 | -1.81, 1.61 |  | -1.03 | -2.13, 0.08 |
|  |  | quantile 3 | 0.29 | -1.41, 2.00 |  | -1.39 | -2.58, -0.20 |
|  |  | quantile 4 | -0.97 | -3.05, 1.12 |  | -3.45 | -4.70, -2.21 |
|  | Women |  |  |  |  |  |  |
|  |  | quantile 1 | (reference) |  |  | (reference) |  |
|  |  | quantile 2 | -1.54 | -2.69, -0.39 |  | -0.91 | -1.60, -0.23 |
|  |  | quantile 3 | -1.22 | -2.37, -0.07 |  | -0.98 | -1.72, -0.24 |
|  |  | quantile 4 | -1.64 | -3.00, -0.28 |  | -1.79 | -2.58, -1.00 |
| Breast cancer |  |  |  |  |  |  |  |
|  | Women |  |  |  |  |  |  |
|  |  | quantile 1 | (reference) |  |  | (reference) |  |
|  |  | quantile 2 | 0.45 | -5.73, 6.63 |  | 5.11 | 0.56, 9.65 |
|  |  | quantile 3 | 1.12 | -5.04, 7.29 |  | 0.18 | -4.73, 5.09 |
|  |  | quantile 4 | -14.36 | -21.65, -7.06 |  | -0.60 | -5.81, 4.62 |

| Morbidity | Sex | SES  quantile | Education level model^e^ | |  | Employment rate model^f^ | |
| --- | --- | --- | --- | --- | --- | --- | --- |
|  |  |  | β | 95% CI |  | β | 95% CI |
| Lung cancer |  |  |  |  |  |  |  |
|  | Men |  |  |  |  |  |  |
|  |  | quantile 1 | (reference) |  |  | (reference) |  |
|  |  | quantile 2 | -3.83 | -8.04, 0.37 |  | 1.74 | -2.40, 5.90 |
|  |  | quantile 3 | -0.64 | -4.90, 3.61 |  | -1.92 | -6.57, 2.73 |
|  |  | quantile 4 | -6.62 | -10.86, -2.38 |  | -5.84 | -11.19, -0.49 |
|  | Women |  |  |  |  |  |  |
|  |  | quantile 1 | (reference) |  |  | (reference) |  |
|  |  | quantile 2 | 0.59 | -1.67, 2.85 |  | -3.44 | -3.53, 0.70 |
|  |  | quantile 3 | 2.26 | -0.05, 4.57 |  | -5.28 | -4.62, -0.15 |
|  |  | quantile 4 | 0.15 | -2.17, 2.47 |  | -1.20 | -3.84, 1.75 |
| Stomach cancer |  |  |  |  |  |  |  |
|  | Men |  |  |  |  |  |  |
|  |  | quantile 1 | (reference) |  |  | (reference) |  |
|  |  | quantile 2 | 7.71 | 2.29, 13.13 |  | 0.72 | -4.66, 6.10 |
|  |  | quantile 3 | 5.01 | -0.47, 10.50 |  | -2.70 | -8.72, 3.32 |
|  |  | quantile 4 | -2.03 | -7.50, 3.44 |  | -6.32 | -13.25, 0.61 |
|  | Women |  |  |  |  |  |  |
|  |  | quantile 1 | (reference) |  |  | (reference) |  |
|  |  | quantile 2 | 1.85 | -1.30, 5.00 |  | -1.41 | -6.40, -0.47 |
|  |  | quantile 3 | 0.70 | -2.53, 3.92 |  | -2.38 | -8.41, -2.14 |
|  |  | quantile 4 | -1.73 | -4.96, 1.51 |  | -1.05 | -5.12, 2.72 |
| Colorectal cancer |  |  |  |  |  |  |  |
|  | Men |  |  |  |  |  |  |
|  |  | quantile 1 | (reference) |  |  | (reference) |  |
|  |  | quantile 2 | 17.95 | 11.77, 24.12 |  | -1.84 | -8.05, 4.37 |
|  |  | quantile 3 | 17.80 | 11.55, 24.05 |  | -8.16 | -15.10, -1.21 |
|  |  | quantile 4 | 6.09 | -0.13, 12.32 |  | -13.56 | -21.59, -5.59 |
|  | Women |  |  |  |  |  |  |
|  |  | quantile 1 | (reference) |  |  | (reference) |  |
|  |  | quantile 2 | 8.96 | 4.71, 13.21 |  | -3.74 | -7.75, 0.27 |
|  |  | quantile 3 | 8.28 | 3.93, 12.63 |  | -12.31 | -16.55, -8.07 |
|  |  | quantile 4 | 3.08 | -1.29, 7.44 |  | -8.38 | -13.68, -3.07 |
| Liver cancer |  |  |  |  |  |  |  |
|  | Men |  |  |  |  |  |  |
|  |  | quantile 1 | (reference) |  |  | (reference) |  |
|  |  | quantile 2 | 0.384 | -1.09, 1.86 |  | -0.26 | -1.72, 1.20 |
|  |  | quantile 3 | -0.46 | -1.96, 1.03 |  | -0.01 | -1.65, 1.63 |
|  |  | quantile 4 | -1.93 | -3.42, -0.44 |  | -2.42 | -4.31, -0.53 |
|  | Women |  |  |  |  |  |  |
|  |  | quantile 1 | (reference) |  |  | (reference) |  |
|  |  | quantile 2 | -0.73 | -1.72, 0.27 |  | -0.66 | -1.58, 0.27 |
|  |  | quantile 3 | -0.47 | -1.49, 0.55 |  | -0.41 | -1.39, 0.57 |
|  |  | quantile 4 | -1.64 | -2.66, -0.61 |  | 0.67 | -0.55, 1.90 |
| Breast cancer |  |  |  |  |  |  |  |
|  | Women |  |  |  |  |  |  |
|  |  | quantile 1 | (reference) |  |  | (reference) |  |
|  |  | quantile 2 | 4.34 | -1.14, 9.81 |  | -5.61 | -10.76, -0.46 |
|  |  | quantile 3 | 4.78 | -0.82, 10.38 |  | -3.83 | -9.28, 1.62 |
|  |  | quantile 4 | 1.51 | -4.11, 7.13 |  | -5.01 | -11.82, 1.80 |

| Mortality | Sex | SES  quantile | Land price model | |  | Neighborhood income model | |
| --- | --- | --- | --- | --- | --- | --- | --- |
|  |  |  | β | 95% CI |  | β | 95% CI |
| Lung cancer |  |  |  |  |  |  |  |
|  | Men |  |  |  |  |  |  |
|  |  | quantile 1 | (reference) |  |  | (reference) |  |
|  |  | quantile 2 | -1.88 | -5.81, 2.05 |  | 1.01 | -1.32, 3.33 |
|  |  | quantile 3 | -2.36 | -6.28, 1.56 |  | -1.18 | -3.68, 1.31 |
|  |  | quantile 4 | -5.84 | -10.63, -1.05 |  | -4.75 | -7.37, -2.13 |
|  | Women |  |  |  |  |  |  |
|  |  | quantile 1 | (reference) |  |  | (reference) |  |
|  |  | quantile 2 | -0.36 | -2.40, 1.68 |  | 1.71 | 0.51, 2.91 |
|  |  | quantile 3 | -0.12 | -2.15, 1.92 |  | 0.06 | -1.24, 1.36 |
|  |  | quantile 4 | -0.32 | -2.73, 2.09 |  | -0.57 | -1.94, 0.81 |
| Stomach cancer |  |  |  |  |  |  |  |
|  | Men |  |  |  |  |  |  |
|  |  | quantile 1 | (reference) |  |  | (reference) |  |
|  |  | quantile 2 | 0.41 | -2.35, 3.17 |  | 0.04 | -1.81, 1.90 |
|  |  | quantile 3 | -1.13 | -3.88, 1.62 |  | -1.57 | -3.56, 0.42 |
|  |  | quantile 4 | -4.65 | -8.02, -1.29 |  | -3.73 | -5.82, -1.64 |
|  | Women |  |  |  |  |  |  |
|  |  | quantile 1 | (reference) |  |  | (reference) |  |
|  |  | quantile 2 | 1.39 | -0.15, 2.93 |  | -0.49 | -1.58, 0.60 |
|  |  | quantile 3 | 0.42 | -1.16, 1.96 |  | -0.56 | -1.73, 0.62 |
|  |  | quantile 4 | -1.48 | -3.30, 0.33 |  | -1.81 | -3.06, -0.56 |
| Colorectal cancer |  |  |  |  |  |  |  |
|  | Men |  |  |  |  |  |  |
|  |  | quantile 1 | (reference) |  |  | (reference) |  |
|  |  | quantile 2 | -1.97 | -4.55, 0.61 |  | -0.88 | -2.54, 0.78 |
|  |  | quantile 3 | -2.37 | -4.95, 0.20 |  | -2.94 | -4.72, -1.16 |
|  |  | quantile 4 | -4.00 | -7.14, -0.85 |  | -5.21 | -7.08, -3.34 |
|  | Women |  |  |  |  |  |  |
|  |  | quantile 1 | (reference) |  |  | (reference) |  |
|  |  | quantile 2 | 0.53 | -1.38, 2.44 |  | 0.38 | -0.89, 1.64 |
|  |  | quantile 3 | 0.86 | -1.05, 2.77 |  | 0.04 | -1.33, 1.40 |
|  |  | quantile 4 | -1.30 | -3.56, 0.96 |  | 1.70 | -3.16, -0.25 |
| Liver cancer |  |  |  |  |  |  |  |
|  | Men |  |  |  |  |  |  |
|  |  | quantile 1 | (reference) |  |  | (reference) |  |
|  |  | quantile 2 | -0.40 | -1.72, 0.92 |  | -0.55 | -1.37, 0.26 |
|  |  | quantile 3 | -0.28 | -1.60, 1.04 |  | -1.05 | -1.93, -0.17 |
|  |  | quantile 4 | -0.88 | -2.49, 0.73 |  | -2.15 | -3.07, -1.23 |
|  | Women |  |  |  |  |  |  |
|  |  | quantile 1 | (reference) |  |  | (reference) |  |
|  |  | quantile 2 | 0.25 | -0.52, 1.03 |  | -0.54 | -1.02, -0.06 |
|  |  | quantile 3 | 0.29 | -0.48, 1.06 |  | -0.63 | -1.14, -0.11 |
|  |  | quantile 4 | 0.07 | -0.85, 0.98 |  | -1.41 | -1.96, -0.86 |
| Breast cancer |  |  |  |  |  |  |  |
|  | Women |  |  |  |  |  |  |
|  |  | quantile 1 | (reference) |  |  | (reference) |  |
|  |  | quantile 2 | 1.73 | 0.21, 3.26 |  | -0.01 | -1.10, 1.08 |
|  |  | quantile 3 | 2.29 | 0.77, 3.81 |  | -0.62 | -1.80, 0.55 |
|  |  | quantile 4 | -0.33 | -2.13, 1.47 |  | -0.96 | -2.21, 0.29 |

| Mortality | Sex | SES  quantile | Education level model | |  | Employment rate model | |
| --- | --- | --- | --- | --- | --- | --- | --- |
|  |  |  | β | 95% CI |  | β | 95% CI |
| Lung cancer |  |  |  |  |  |  |  |
|  | Men |  |  |  |  |  |  |
|  |  | quantile 1 | (reference) |  |  | (reference) |  |
|  |  | quantile 2 | -3.55 | -6.95, -0.15 |  | -0.71 | -4.09, 2.68 |
|  |  | quantile 3 | -1.97 | -5.41, 1.47 |  | -1.72 | -5.51, 2.07 |
|  |  | quantile 4 | -6.25 | -9.68, -2.82 |  | -3.63 | -8.00, 0.73 |
|  | Women |  |  |  |  |  |  |
|  |  | quantile 1 | (reference) |  |  | (reference) |  |
|  |  | quantile 2 | -1.35 | -3.11, 0.41 |  | -1.67 | -3.32, -0.02 |
|  |  | quantile 3 | -0.21 | -2.02, 1.59 |  | -2.44 | -4.18, -0.70 |
|  |  | quantile 4 | -1.74 | -3.55, 0.07 |  | -0.71 | -2.88, 1.47 |
| Stomach cancer |  |  |  |  |  |  |  |
|  | Men |  |  |  |  |  |  |
|  |  | quantile 1 | (reference) |  |  | (reference) |  |
|  |  | quantile 2 | 2.43 | 0.02, 4.84 |  | 0.77 | -1.61, 3.14 |
|  |  | quantile 3 | 0.55 | -1.89, 2.98 |  | -0.12 | -2.78, 2.54 |
|  |  | quantile 4 | -1.76 | -4.18, 0.67 |  | -3.37 | -6.43, -0.31 |
|  | Women |  |  |  |  |  |  |
|  |  | quantile 1 | (reference) |  |  | (reference) |  |
|  |  | quantile 2 | 1.63 | 0.30, 2.97 |  | -0.46 | -1.73, 0.81 |
|  |  | quantile 3 | 0.51 | -0.85, 1.88 |  | -1.42 | -2.76, -0.07 |
|  |  | quantile 4 | -0.42 | -1.80, 0.95 |  | -0.46 | -2.14, 1.22 |
| Colorectal cancer |  |  |  |  |  |  |  |
|  | Men |  |  |  |  |  |  |
|  |  | quantile 1 | (reference) |  |  | (reference) |  |
|  |  | quantile 2 | 1.80 | -0.43, 4.03 |  | -0.33 | -2.55, 1.89 |
|  |  | quantile 3 | -0.19 | -2.44, 2.07 |  | -2.26 | -4.75, 0.22 |
|  |  | quantile 4 | -3.29 | -5.53, -1.03 |  | -3.01 | -5.87, -0.15 |
|  | Women |  |  |  |  |  |  |
|  |  | quantile 1 | (reference) |  |  | (reference) |  |
|  |  | quantile 2 | 2.23 | 0.56, 3.89 |  | -1.08 | -2.64, 0.49 |
|  |  | quantile 3 | 1.05 | -0.65, 2.75 |  | -1.94 | -3.59, -0.28 |
|  |  | quantile 4 | 0.56 | -1.15, 2.27 |  | -1.44 | -3.51, 0.63 |
| Liver cancer |  |  |  |  |  |  |  |
|  | Men |  |  |  |  |  |  |
|  |  | quantile 1 | (reference) |  |  | (reference) |  |
|  |  | quantile 2 | 0.07 | -1.07, 1.22 |  | -0.55 | -1.68, 0.59 |
|  |  | quantile 3 | -0.77 | -1.93, 0.39 |  | -1.26 | -2.53, 0.01 |
|  |  | quantile 4 | -1.58 | -2.74, -0.43 |  | -1.62 | -3.09, -0.16 |
|  | Women |  |  |  |  |  |  |
|  |  | quantile 1 | (reference) |  |  | (reference) |  |
|  |  | quantile 2 | 0.04 | -0.63, 0.71 |  | -0.55 | -1.17, 0.07 |
|  |  | quantile 3 | 0.07 | -0.61, 0.76 |  | -0.66 | -1.32, -0.01 |
|  |  | quantile 4 | -0.68 | -1.37, 0.01 |  | -0.83 | -1.66, -0.01 |
| Breast cancer |  |  |  |  |  |  |  |
|  | Women |  |  |  |  |  |  |
|  |  | quantile 1 | (reference) |  |  | (reference) |  |
|  |  | quantile 2 | 1.18 | -0.15, 2.51 |  | -1.41 | -2.66, -0.17 |
|  |  | quantile 3 | 0.57 | -0.80, 1.93 |  | -1.77 | -3.09, -0.45 |
|  |  | quantile 4 | 0.08 | -1.29, 1.45 |  | -3.36 | -5.01, -1.71 |

a Group divided into quartiles from the lowest community SES. Quantile 1 is the group with the lowest community SES.

b Linear regression, morbidity or mortality ~ land price + year + aging rate

c Linear regression, morbidity or mortality ~ neighborhood income + year + aging rate

d Regression coefficient of community SES indicator

e Linear regression, morbidity or mortality ~ education level + year + aging rate

f Linear regression, morbidity or mortality ~ employment rate + year + aging rate

SES indicates socioeconomic status; CI, confidence interval.
