# Supplementary material for: Association between socioeconomic background and cancer: An ecological study using cancer registry and various community socioeconomic status indicators in Kanagawa, Japan
Source: PLoS One. 2025 Jul 9;20(7):e0326895. doi: 10.1371/journal.pone.0326895 (PMC12240336; doi:10.1371/journal.pone.0326895)
Supplement: S1 Data — S1 File. Community SES information. S1 Fig. Scatterplot of the relationship between community land price (A), neighborhood income (B), education level (C), and employment rate (D), with stomach cancer morbidity and mortality for men and women in Kanagawa, Japan, 2000–2015. Each plot shows data per year and community. 1$ = 133 Japanese Yen, the rate on March 20, 2023. S2 Fig. Scatterplot of the relationship between community land price (A), neighborhood income (B), education level (C), and employment rate (D), with colorectal cancer morbidity and mortality for men and women in Kanagawa, Japan, 2000–2015. Each plot shows data per year and community. 1$ = 133 Japanese Yen, the rate on March 20, 2023. S3 Fig. Scatterplot of the relationship between community land price (A), neighborhood income (B), education level (C), and employment rate (D), with liver cancer morbidity and mortality for men and women in Kanagawa, Japan, 2000–2015. Each plot shows data per year and community. 1$ = 133 Japanese Yen, the rate on March 20, 2023. S4 Fig. Scatterplot of the relationship between community land price (A), neighborhood income (B), education level (C), and employment rate (D), with breast cancer morbidity and mortality for women in Kanagawa, Japan, 2000–2015. Each plot shows data per year and community. 1$ = 133 Japanese Yen, the rate on March 20, 2023. S1 Table. Correlation coefficients of the aging rate, screening rate, and community SES indicators in Kanagawa, Japan, 2000–2015. S2 Table. VIF of the Poisson regression using community SES indicator, aging rate, and year as explanatory variables. S3 Table. VIF of the Poisson regression using community SES indicator, aging rate, year, and municipality code as explanatory variables. S4 Table. Multilevel analysis by the year for cancer morbidity in Kanagawa, Japan, 2000–2015. S5 Table. Multilevel analysis by the year for cancer mortality in Kanagawa, Japan, 2000–2015. S6 Table. Multilevel analysis by the municipality code for canc [file pone.0326895.s001.zip › S5_Table.docx]

**S5 Table. Multilevel analysis by the year for cancer mortality in Kanagawa, Japan, 2000-2015.**

| Mortality | Sex | β^a^ (Land price model^b^) | |  | β (Neighborhood income model^c^) | |
| --- | --- | --- | --- | --- | --- | --- |
|  |  | FE | RE variance |  | FE | RE variance |
| Lung cancer |  |  |  |  |  |  |
|  | Men | -1.81 | 4.6 |  | -2.32 | 4.0 |
|  | Women | 1.84 | 3.6 |  | -0.50 | <0.1 |
| Stomach cancer |  |  |  |  |  |  |
|  | Men | -1.06 | 9.0 |  | -1.46 | <0.1 |
|  | Women | 0.32 | 2.0 |  | -0.27 | <0.1 |
| Colorectal cancer |  |  |  |  |  |  |
|  | Men | -1.80 | 84.1 |  | -2.36 | 1.2 |
|  | Women | 1.26 | 0.6 |  | -1.41 | 2.9 |
| Liver cancer |  |  |  |  |  |  |
|  | Men | 1.13 | <0.1 |  | -0.85 | <0.1 |
|  | Women | 0.15 | <0.1 |  | -0.87 | 0.6 |
| Breast cancer |  |  |  |  |  |  |
|  | Women | 1.73 | 1.9 |  | -1.51 | 1.4 |

| Mortality | Sex | β (Education level model^d^) | |  | β (Employment rate model^e^) | |
| --- | --- | --- | --- | --- | --- | --- |
|  |  | FE | RE variance |  | FE | RE variance |
| Lung cancer |  |  |  |  |  |  |
|  | Men | -1.39 | <0.1 |  | -2.18 | <0.1 |
|  | Women | 0.69 | <0.1 |  | -2.57 | 7.2 |
| Stomach cancer |  |  |  |  |  |  |
|  | Men | -0.16 | <0.1 |  | -2.26 | <0.1 |
|  | Women | -0.85 | 1.0 |  | -2.52 | 14.2 |
| Colorectal cancer |  |  |  |  |  |  |
|  | Men | -1.85 | 7.3 |  | -1.48 | <0.1 |
|  | Women | 0.78 | <0.1 |  | -4.71 | <0.1 |
| Liver cancer |  |  |  |  |  |  |
|  | Men | -0.27 | <0.1 |  | -1.61 | <0.1 |
|  | Women | -0.01 | <0.1 |  | -1.94 | 3.6 |
| Breast cancer |  |  |  |  |  |  |
|  | Women | 0.49 | <0.1 |  | -5.94 | 24.8 |

a Regression coefficient of community SES indicator calculated by the multilevel analysis

b Multilevel model by year, mortality ~ land price + year + aging rate

c Multilevel model by year, mortality ~ neighborhood income + year + aging rate

d Multilevel model by year, mortality ~ education level + year + aging rate

e Multilevel model by year, mortality ~ employment rate + year + aging rate

FE indicates Fixed effect; RF, Random effect.
