# Supplementary material for: Association between socioeconomic background and cancer: An ecological study using cancer registry and various community socioeconomic status indicators in Kanagawa, Japan
Source: PLoS One. 2025 Jul 9;20(7):e0326895. doi: 10.1371/journal.pone.0326895 (PMC12240336; doi:10.1371/journal.pone.0326895)
Supplement: S1 Data — S1 File. Community SES information. S1 Fig. Scatterplot of the relationship between community land price (A), neighborhood income (B), education level (C), and employment rate (D), with stomach cancer morbidity and mortality for men and women in Kanagawa, Japan, 2000–2015. Each plot shows data per year and community. 1$ = 133 Japanese Yen, the rate on March 20, 2023. S2 Fig. Scatterplot of the relationship between community land price (A), neighborhood income (B), education level (C), and employment rate (D), with colorectal cancer morbidity and mortality for men and women in Kanagawa, Japan, 2000–2015. Each plot shows data per year and community. 1$ = 133 Japanese Yen, the rate on March 20, 2023. S3 Fig. Scatterplot of the relationship between community land price (A), neighborhood income (B), education level (C), and employment rate (D), with liver cancer morbidity and mortality for men and women in Kanagawa, Japan, 2000–2015. Each plot shows data per year and community. 1$ = 133 Japanese Yen, the rate on March 20, 2023. S4 Fig. Scatterplot of the relationship between community land price (A), neighborhood income (B), education level (C), and employment rate (D), with breast cancer morbidity and mortality for women in Kanagawa, Japan, 2000–2015. Each plot shows data per year and community. 1$ = 133 Japanese Yen, the rate on March 20, 2023. S1 Table. Correlation coefficients of the aging rate, screening rate, and community SES indicators in Kanagawa, Japan, 2000–2015. S2 Table. VIF of the Poisson regression using community SES indicator, aging rate, and year as explanatory variables. S3 Table. VIF of the Poisson regression using community SES indicator, aging rate, year, and municipality code as explanatory variables. S4 Table. Multilevel analysis by the year for cancer morbidity in Kanagawa, Japan, 2000–2015. S5 Table. Multilevel analysis by the year for cancer mortality in Kanagawa, Japan, 2000–2015. S6 Table. Multilevel analysis by the municipality code for canc [file pone.0326895.s001.zip › S9_Table.docx]

**S9 Table. Average and SD of land price, neighborhood income, education level, employment rate, morbidity, and mortality for each cancer type in urban, town, and rural areas in Kanagawa, Japan, 2000-2015.**

| **Characteristics** | Urban^g^ | *P* value  Urban-Town^h^ | Town^i^ | *P* value  Town-Rural^j^ | Rural^k^ | *P* value  Rural-Urban^l^ |
| --- | --- | --- | --- | --- | --- | --- |
| Average population (SD), ×10^3^ | 3376(1260) |  | 179(111) |  | 22(13) |  |
|  |  |  |  |  |  |  |
| Average land price (SD), $×10^3^/m^2a^ | 2.1(0.8) | <0.01 | 1.5(0.5) | <0.01 | 0.9(0.4) | <0.01 |
| Average neighborhood income (SD), $×10^3b^ | 42.5(4.8) | <0.01 | 40.3(4.0) | 0.95 | 40.0(4.5) | 0.02 |
| Average education level (SD), %^c^ | 40.9(7.3) | <0.01 | 35.6(7.8) | 0.02 | 30.2(8.4) | <0.01 |
| Average employment rate (SD), %^d^ | 95.1(1.1) | 0.25 | 94.8(1.0) | 0.10 | 95.2(1.2) | 0.73 |
|  |  |  |  |  |  |  |
| Average morbidity, number of persons/population(SD)^e^ |  |  |  |  |  |  |
| Lung cancer | 33.5(19.4) | 0.34 | 36.7(21.0) | 0.84 | 39.2(22.8) | 0.13 |
| Stomach cancer | 43.8(22.5) | 0.08 | 49.9(23.1) | 0.80 | 50.6(25.7) | 0.02 |
| Colorectal cancer | 80.4(40.0) | 0.91 | 82.8(41.5) | 0.33 | 79.7(46.6) | 0.47 |
| Liver cancer | 7.0(4.1) | 0.97 | 7.1(4.8) | 0.39 | 9.1(6.91) | 0.44 |
| Breast cancer | 90.8(42.8) | 0.82 | 92.5(43.3) | 0.84 | 91.9(47.4) | 0.99 |
|  |  |  |  |  |  |  |
| Average Mortality, number of persons/population(SD)^f^ |  |  |  |  |  |  |
| Lung cancer | 23.1(15.1) | 0.47 | 25.3(17.1) | 0.99 | 27.5(20.6) | 0.42 |
| Stomach cancer | 15.7(8.6) | 0.47 | 18.6(9.8) | 0.58 | 21.7(12.7) | <0.01 |
| Colorectal cancer | 23.6(17.7) | 0.81 | 25.6(19.6) | 0.81 | 30.6(26.3) | 0.99 |
| Liver cancer | 4.7(3.5) | 0.94 | 4.7(3.9) | 0.01 | 6.4(6.5) | <0.01 |
| Breast cancer | 14.6(9.2) | 0.87 | 15.1(8.9) | <0.01 | 14.9(9.1) | <0.01 |

a Average land price and SD in urban, town, and rural areas, 1$ = 133 Japanese Yen, the rate on March 20^th^, 2023)

b Average neighborhood income and SD in urban, town, and rural areas, 1$ = 133 Japanese Yen, the rate on March 20^th^, 2023)

c Average education level and SD in urban, town, and rural areas

d Average employment rate and SD in urban, town, and rural areas

e Average morbidity of each cancer type per 100 000 people in urban, town, and rural areas

f Average mortality of each cancer type per 100 000 people in urban, town, and rural areas

g Municipalities with more than 500 000 people (community SES, morbidity, and mortality were calculated on a ward-by-ward basis)

h *P*-value of ANOVA between urban and town areas for each variable

i Municipalities with 50,000 to 500 000 people

j *P*-value of ANOVA between town and rural areas for each variable

k Municipalities with fewer than 50 000 people

l *P*-value of ANOVA between rural and urban areas for each variable

SES indicates socioeconomic status.
