# Supplementary material for: Association between socioeconomic background and cancer: An ecological study using cancer registry and various community socioeconomic status indicators in Kanagawa, Japan
Source: PLoS One. 2025 Jul 9;20(7):e0326895. doi: 10.1371/journal.pone.0326895 (PMC12240336; doi:10.1371/journal.pone.0326895)
Supplement: Attachment — Submitted filename: Response to Reviewers.docx [file pone.0326895.s003.docx]

Journal Requirements:

1. When submitting your revision, we need you to address these additional requirements.

Please ensure that your manuscript meets PLOS ONE's style requirements, including those for file naming. The PLOS ONE style templates can be found at

https://journals.plos.org/plosone/s/file?id=wjVg/PLOSOne_formatting_sample_main_body.pdf and

<https://journals.plos.org/plosone/s/file?id=ba62/PLOSOne_formatting_sample_title_authors_affiliations.pdf>

Reply:

Thank you for your comment. We revised our manuscript according to the style requirements.

2. We noted in your submission details that a portion of your manuscript may have been presented or published elsewhere. “This manuscript is a part of a PhD dissertation, and its abstract will be available on the Yamagata University website at https://yamagata.repo.nii.ac.jp/?page=1&size=20&sort=-pyear&search_type=0&q=0.”

Please clarify whether this [conference proceeding or publication] was peer-reviewed and formally published. If this work was previously peer-reviewed and published, in the cover letter please provide the reason that this work does not constitute dual publication and should be included in the current manuscript.

Reply:

Thank you for your comment. This was an outline, not a peer-reviewed, formally published paper, so it does not constitute double publication.

3. When completing the data availability statement of the submission form, you indicated that you will make your data available on acceptance. We strongly recommend all authors decide on a data sharing plan before acceptance, as the process can be lengthy and hold up publication timelines. Please note that, though access restrictions are acceptable now, your entire data will need to be made freely accessible if your manuscript is accepted for publication. This policy applies to all data except where public deposition would breach compliance with the protocol approved by your research ethics board. If you are unable to adhere to our open data policy, please kindly revise your statement to explain your reasoning and we will seek the editor's input on an exemption. Please be assured that, once you have provided your new statement, the assessment of your exemption will not hold up the peer review process.

Reply:

Thank you for your comment. The cancer data set used in this study cannot be made publicly available. We have revised our data availability statement as follows:

The cancer registry data was provided by Kanagawa Prefecture with permission of the research use. The guideline of the Population-based Cancer Registry of limited the data usage within the applicants, thus the data set used in this study cannot be set publicly available. Kanagawa Prefecture in Japan can provide the data for all interested researchers after the accept of the application for the data usage. The contact email address is kikaku@gancen.asahi.yokohama.jp.

-Additional Editor Comments:

Thank you for submitting the manuscript to PLOS ONE.

As the reviewers commented, we consider that the Methods section needs more explanation.

A detailed explanation how you analysed your data and a justification of why you used the method are necessary so that the readers can replicate the results.

Regarding the Results section, there seems to be no description of the results of Table 2 and 3.

People can read tables, but the authors still need to summarise and explain what the numbers in the tables mean.

For the Discussion, the manuscript is difficult to follow. Please refer to an academic writing book to understand how it is usually structured.

Reply:

Thank you for your suggestion.

We made revisions with your suggestion for methods when responding to the points raised by “Reviewer#1 Major points Materials and Methods 1)”, “Reviewer#1 Major points Materials and Methods 3)”, “Reviewer#1 Minor points 3”, “Reviewer#2 4”, “Reviewer#2 6”, and “Reviewer#2 3”.

Also, we made revisions with your suggestion for Results when responding to the points raised by “Reviewer#1 Minor points 5”, “Reviewer#1 Minor points 7”, “Reviewer#2 12”.

Furthermore, we made revisions with your suggestion for discussion when responding to the points raised by “Reviewer#1 Major points Discussion 2)”, “Reviewer#1 Major points Discussion 3)”, “Reviewer#1 Major points Discussion 8)”

Reviewer #1

Major points

Introduction

-1) Page4: L88-90

The authors described that many studies used comprehensive indicators. However, I think there is not enough explanation as to why many previous studies used comprehensive indicators instead of each community-level factor, what can be ascertained by comprehensive indicators, and then why only used comprehensive indicators were not enough. Please review the characteristics of Areal Deprivation Index by Prof. Nakaya or some comprehensive indicators used in other countries and explain more detailed the issue with comprehensive indicators about community-level socioeconomic status.

Reply:

Thank you for your comment. We have added the following sentence:

Line 80: Comprehensive indicators have had the advantage of being able to reflect the overlap in deprivation across various life domains [17]. However, consensus on the choice of comprehensive indicators and their weighting is lacking [17]. A previous study demonstrated that some comprehensive indicators were incompatible with each other and care should be given when selecting comprehensive indicators for research use [20], as the relationship with cancer could differ depending on the comprehensive indicator used. ~~However~~ Furthermore,

Line 97: In addition, the use of a single community SES indicator could eliminate the issues with generalization of weights, a challenge associated with a comprehensive community SES indicator. The approach of this paper was considered to facilitate application to other regions.

-2) Page4: L90-93

The authors pointed out the challenge about the different stage of life course that community-level SES affect people. However, this present study did not examine the impact of differences in the time points of community-level SES on cancer incidence and mortality. If the authors pointed out two challenges not only the comprehensive indicators but also life course, I think the authors would prefer to analysis the time trends or changes of time points about community-level SES. Alternatively, if the authors only pointed out this issue and have a reason for not focusing on it, please explain.

Reply:

Thank you for your comment. We have added another analysis and the following sentence:

Line 208: Other analyses of correlation coefficients between community SES and year were conducted to confirm changes in SES over time.

Line 276: The results of the correlation coefficient, VIF of the Poisson regression, multilevel analysis, t-test by population size, Linear regression using quartiles of community SES, morbidity and mortality for each year, the number of deaths for each type of cancer registered in the vital statistics, and the correlation coefficient using age-adjusted mortality as response variables are shown in S1–S12 Tables.

Line 488: Given that there were likely to be changes in SES over time (S8 Table), and given the possibility that the impact of SES on cancer might vary over the life course [21,22], it was still possible that changes in SES over time affect cancer incidence and mortality, and this is a topic for future research.

Materials and Methods

-1) Page5: L126-128

I think the explanation about the calculation of cancer incidence and cancer mortality was insufficient. It is not described what data on population by sex, year, and municipality was used for the denominator. Citation in No. 24 is a link to the e-stat top page, with no details of the data source. Please state the data source and cite the link to the correct data source.

Reply:

Thank you for your suggestion. We have revised the reference information in S1 file, as the comment of 4 from Reviewer#2, and as follow:

Line 669: 26. ~~Neighborhood income, education level, employment rate, population, and aging rate information in Kanagawa, Japan. [Cited 2024 April 20]. Available from:https://www.e-stat.go.jp/.~~ Population information in Kanagawa, Japan. [Cited 2024 August 30]. Available from: <https://www.e-stat.go.jp/stat-search/database?page=1&toukei=00200524&tstat=000000090001>.

Line 672: 28. The number of people over 65 years old in Kanagawa, Japan. [Cited 2024 August 30]. Available from: https://www.e-stat.go.jp/stat-search/database?page=1&toukei=00200521&tstat=000001080615.

-2) Page5-6: L 130-132

The authors mentioned that the authors took an average to consider for outliers, but why did the authors not divide the data for each municipality calculated every five years into quintiles based on community-level SES? I have concern that if the authors take an average, it becomes a representative value for five years, which seems to reduce the amount of information.

Reply:

Thank you for your suggestion. We have performed linear regressions using morbidity and mortality rates for each year. In this regression, since data were only available for education levels every 10 years and income and employment rates every 5 years, data for the nearest year were substituted for the years in which those data were missing, and the community SES were grouped into quartiles. However, because of such quartiles and the substitution of community SES, differences in morbidity and mortality due to differences in community SES might have disappeared. Therefore, we have added this analysis in supporting information (S10_Table) and the following sentence:

Line 213: To test the effect of the loss of information due to the calculation of morbidity and mortality rates as 5-year averages, linear regressions were performed using morbidity and mortality rates for each year. In this regression, the community SES values available for every 5 or 10 years were used for the other years as well, and the community SES were grouped into quartiles

Line 276: The results of the correlation coefficient, VIF of the Poisson regression, multilevel analysis, t-test by population size, Linear regression using quartiles of community SES, morbidity and mortality for each year, and the correlation coefficient using age-adjusted mortality as response variables are shown in S1–S12 Tables.

Line 501: Linear regressions using quadratized community SES and morbidity and mortality rates for each year showed significantly lower morbidity and mortality rates in the fourth quartile than in the first quartile in the 7, 14, 6, and 9 models for land price, income, education level, and employment rate, respectively (S10 Table). Despite the use of unaveraged morbidity and mortality rates, ~~compared to Tables 2 and 3,~~ the proportion of models in which the response variable tends to decrease with increasing SES was reduced compared to the models using averaged morbidity and mortality. This might be due to the quartiles of community SES and the fact that community SES values that could only be used every 5 or 10 years were substituted for other years.

-3) Page6: L 136-137

The statement of the rationale for the choice of why these SES indicators were used is missing. In addition, the reason for focused on cancer type: lung, stomach, colorectal, liver, and breast is missing. Furthermore, as with population, the authors cited only the top page of e-stat, without a description of the data source for each factor and the year used in this study. The current description seems to make it difficult to replicate this study, so I think a more detailed description of the method is needed

Reply:

Thank you for your comment. We have selected these SES indicators to cover the whole person's life and added the specific point in a person's life that these SES indicators reflected and detailed calculation methods references information in S1 File, as Reviewer#2 4) suggested, and the following explanation:

Line 115: The morbidity and mortality data of patients with cancer were obtained from the Kanagawa Cancer Registry from 2000 to 2016, with permission from Kanagawa prefecture in 2020. We analyzed the data of pathologically diagnosed lung, stomach, colorectal, liver, and breast cancer, ~~from 2000 to 2016, with permission from Kanagawa prefecture in 2020~~ which were among the top five most common causes of cancer deaths by gender in Japan [23] in recent years. Although, for liver cancer, screening was done by testing liver enzymes, these cancers have screening tests that are easy to implement.

-4) Pafe7: L173-174

What was the reason for the decision to analyses all years instead of analyzing each five years separately? Cancer incidence and mortality, and community-level SES seem to have varied over the 15 years period. It may be better to consider analysis that separate by 5-years or include an interaction between community-level SES and year-period.

Reply:

Thank you for your suggestion. As you mentioned, cancer incidence and mortality, and community SES have changed over time. Therefore, we added years as an explanatory variable in the multivariate analysis considering the effect of year. We also considered analysis to separate by 5 years using multilevel analysis (S4, S5 Tables). We have added the following explanations:

Line 198: We also used the year and population rate over 65 years of age (aging rate) [31] in each community as explanatory variables because the year and aging rates may be associated with SES and cancer incidence and death.

Results

-1) Page7: L208-209

Aggregation of cancer incidence and mortality by urban/rural appeared in Supplementary table8, however, there is no description of the method and result about this table at all in this manuscript. As long as the result were presented as supplementary table. Please add as explanation in this manuscript. In addition, why did the authors not include the analysis the urban/rural indicator? As both community-level SES and cancer incidence and mortality tend to vary between urban and rural areas, focusing on this indicator may help to identify high-risk groups of cancer.

Reply:

Thank you for your comment. We have added the following sentence:

Line 209: To identify differences in SES and the number of cancer cases and deaths by population size, the municipalities were divided into three groups according to population size, and t-tests were also conducted.

Line 276: The results of the correlation coefficient, VIF of the Poisson regression, multilevel analysis, t-test by population size, Linear regression using quartiles of community SES, morbidity and mortality for each year, the number of deaths for each type of cancer registered in the vital statistics, and the correlation coefficient using age-adjusted mortality as response variables are shown in S1–S12 Tables.

Line 491: Regarding the analysis by population size, although there were many significant differences between population size and SES, there was little significant association with the number of cancer morbidity, making it difficult to identify those at high risk of cancer morbidity. A previous study predicting the future number of breast cancer patients in Kanagawa Prefecture predicted that the number of breast cancer patients in urban areas would increase with the aging of the population [24]. Therefore, it is possible that in the future, there will be differences in cancer incidence and mortality based on population size. In addition to population size, many other social factors may influence SES and cancer, and the association among these factors, SES, and cancer needs to be further examined.

Discussion

-1) In the Discussion section, it is necessary to be revised to consider the impact of the fact that it was not age-adjusted. Adjustment for ageing rate only cannot consider the differences in the age distribution of cancer incidence and mortality. It would be necessary to consider how the differences in age distribution by municipality affect the association observed between community-level SES and incidence and mortality by cancer type.

Reply:

Thank you for your comment. We added the following sentence:

Line 554: Finally, we could not use age-stratified population data by municipality and year, and thus, we could not calculate the age-adjusted morbidity and mortality. A negative association between community SES and aging rates has been shown (S1 Table), and residual confounding may still occur even with adding year as an explanatory variable.

-2) Page13: L266-268

There seems to be no mention of why there were differences in community-level SES associated with incidence and mortality by cancer type, or why gender differences were observed. Please discuss not only the results that were similar to previous studies, but also the reasons.

Reply:

Thank you for your comment. Because the main theme of this paper is the association between each community SES and cancer, and a description of the association between SES and each cancer type would be too voluminous, we only described the association between liver cancer and SES, which has interesting association with community SES in this paper. As for the gender difference, the number of models that showed negative associations did not differ significantly between men and women, and the description of the associations between each cancer type and each SES for men and women would be too voluminous to mention, so we decided not to mention them in this study. We have added this point as follow:

Line 372: The reason might be that the widening disparity in SES within a country, regardless of country or insurance system, affects cancer incidence and death.

Line 467: Regarding gender differences, the number of models showing negative associations did not differ significantly between men and women. However, land price and education level showed inverse associations with colorectal cancer mortality for men and women. As many statistical models were tested in this study, we cannot rule out the possibility that this result was obtained by chance. However, further validation of these characteristic associations is warranted.

-3) Page15: L 281-283

If each community-level SES is used to identify high-risk strata, what about the challenge of identifying aspects of community-level SES that need to be improved that was pointed out in Introduction? If the focus is on identifying high-risk strata, Comprehensive SES indicator can be used to consider the relation of the factors that constitute the comprehensive SES indicator with respect to each other. The current logic leads to the question: why not just use a comprehensive indicator instead of a factor-specific one? Please explain the authors’ opinion.

Reply:

Thank you for your comment. One of the problems with comprehensive evaluation is how much weight should be given to each factor. We consider this partly because it is difficult to determine whether the weighting can be adapted outside the region where the study was conducted. Although there are still challenges in measuring community SES indicators such as education level, they are more generally applicable to regions other than the one studied than a comprehensive evaluation, and from this reason, we consider that evaluating cancer risk in a region using community SES indicators is more useful than a comprehensive evaluation in this point. We have added this point as follow:

Line 361: Although it may be possible to use a comprehensive evaluation to identify high-risk areas, one significant challenge with a comprehensive evaluation is knowing how much weight each factor should be given. We considered this partly because it was difficult to determine whether the weighting could be adapted outside the region where the study was conducted. Although there are still challenges in measuring community SES indicators such as education level, they are more generally applicable to regions other than the one studied than a comprehensive evaluation; for this reason, we considered that evaluating cancer risk in a region using community SES indicators was more useful than a comprehensive evaluation.

- 4) Page16: L294-295

As the author examined in Supplementary table1, some community-level SES were correlated. Given this result, is it the true identification of the target group to say that the proportion of employment had the greatest impact, and therefore to target areas with the lowest proportion of employment? As I pointed out 3), the consideration of the influence between factors seems to be missing from this Discussion. It may be necessary to conduct an analysis that considers the influence of the factors each other in this study, and then verify whether the influence of the proportion of employment is still significant.

Reply:

Thank you for your suggestion. The models that tested the interaction with all community SES indicators as explanatory variables were considered inappropriate due to the high value of VIF. We have added this point as follow:

Line 201: Additionally, other models that incorporated adding the municipality code as an explanatory variable to account for factors unique to the region beyond the SES were assessed. These models also included all community SES indicators while considering interactions.

Line 281: In the model that tested the interaction with all community SES indicators as explanatory variables, the VIF for land price, neighborhood income, education level, and employment rate were 5.2, 9.0, 7.9, and 7.2 in men and 3.7, 8.9, 6.4, and 3.4 in women, respectively.

Line 518: The models that tested the interaction with all community SES indicators as explanatory variables were considered inappropriate due to the high value of VIF. If the goal is to improve cancer outcomes by improving community SES, we should consider interactions among community SES indicators. However, if the goal is to identify high-risk areas for cancer, then for reasons of simplicity, it would be advantageous to evaluate only a single indicator without considering such interactions.

- 5) Page16: L 313-

With regard to this paragraph, I think it is discussed broadly as the overall community-level SES rather than which community-level SES. Although it is important findings that the association between liver cancer incidence and mortality and community-level SES, the aim of this study was to assess community-level SES by ingle indicator, not comprehensively. Nevertheless, the discussion of the overall community-level SES seems to be out of line with the aim of this study, and I have a question again: why not just use a comprehensive indicator?

In addition, the authors cited previous study, citation No.33, but this study focused on individual-level SES related to hepatitis treatment, which is different from community-level SES that the authors are focusing on this study. A proven association at the individual-level does not necessarily mean that it applies at community level. Please consider presenting evidence not only at individual-level, but also community-level.

Reply:

Thank you for your suggestion. As noted, this paper focused on each community SES, so the following changes were made to focus on income and employment rates, which were found to be particularly useful in the above section:

Line 440: As ~~the community SES indicators,~~ the community neighborhood income and employment rate changed by one standard deviation, the change in morbidity and mortality averaged approximately 0.32 per 100 000 people with liver cancer among the model having significant inverse associations.

We revised the following sentence:

Line 414: Furthermore, ~~a low income was associated with low adherence to hepatitis B treatment~~ countries with a low socioeconomic status had a high burden of hepatitis B [36].

We revised the previous study as follows:

Line 695: 36. ~~Emin Ediz Tütüncü, Rahmet Güner, Yunus Gürbüz, Ayşe Kaya Kalem, Barış Öztürk, İmran Hasanoğlu, et al. Adherence to Nucleoside/Nucleotide Analogue Treatment in Patients with Chronic Hepatitis B. Balkan Med J. 2017;34:540-545.~~ Wang M, Yan L, Wang J, Jin Y, Zheng ZJ. Global burden of hepatitis B attributable to modifiable risk factors from 1990 to 2019: a growing contribution and its association with socioeconomic status. Global Health. 2023;19: 23. doi: [10.1186/s12992-023-00922-z](https://doi.org/10.1186/s12992-023-00922-z).

-6) Page16: L320-321

Screening for hepatitis virus is not an item of the specific health check-up, and the method of receiving this screening varies by municipalities. It seems to be misleading to say that the proportion of specific health check-ups by national health insurance subscribers is low, and therefore the proportion of hepatitis virus screening is also low. The same logic may apply to the H. pylori test, which is the cause of stomach cancer, as some municipalities allow the test to be taken at the same time as the specific health check-ups. The explanation as to why low community-level low SES is related to incidence and mortality in liver cancer does not be clearly. Please reconsider after re-examining the evidence.

Reply:

Thank you for your comment. We meant to mention here that we are screening for liver damage, not hepatitis virus, so we have revised the text as follows:

Line 450: In Japan, where the screening for ~~hepatitis~~ impairment of liver function, aspartate aminotransferase, and alanine aminotransferase is a part of health checkups, having insurance with a high number of low-income members resulted in a low health checkup rate [30]. Other studies in Japan have found that the lower the household income, the less health checkups were taken [35]. That is, in a population that includes a large number of low-income individuals, the individuals are less likely to undergo physical examinations, which may delay the detection of liver damage, leading to more severe hepatitis and the development of liver cancer.

Line 692: 35. Taguchi K, Natsuhara K. Social capital and residents’ consultation behavior for medical checkups and/or cancer screening: research in a provincial area of Japan. [Cited 2024 September 30]. Available from: https://core.ac.uk/outputs/235715128/?source=oai.

-7) Page 17: L 326-328

The interventions currently proposed focus on low SES people living in low SES areas, and not on low SES people living in middle or high SES areas. I suggest the idea of proportionate universalism according to the degree of SES is need. Please confirm proportionate universalism/

(https://health-inequalities.eu/glossary/proportionate-universalism/)

Reply:

Thank you for your suggestion. We have added the following sentence:

Line 428: Significantly positive associations were noted between cancer incidence and mortality for land price and education level. Notably, for colorectal cancer in women, both land price and education level were associated with increased morbidity and mortality. Therefore, implementing targeted colorectal cancer control for women living in areas with higher land prices or education levels may be effective. It is important to consider such individualized cancer control measures in the community SES. One possibility is that the interaction between individual SES and community SES may have increased the risk of further cancer in individuals with low individual SES living in a high community SES due to the interaction. However, because this study was an ecological study and did not measure individual SES, the above possibility could not be verified. Therefore, it is important to verify cancer control measures that focus on various community SES levels.

-8) There is no mention of Strength or Implication of this study in Discussion section. At the end of Introduction, it was stated that “This research could provide insights into the use of community SES in cancer research and cancer control strategies, and lead to more effective cancer control”. In light of this description, please indicate a separate paragraph of implication from the results of this study.

Reply:

Thank you for your comment. We have added the following sentence:

Line 559: The strength of this study was the use of data from the cancer registry, which allowed us to include only those cases with a definite pathological diagnosis of cancer. Another strength was that the Kanagawa Prefecture was the second most populous prefecture in Japan; while the central part of the prefecture was highly urbanized, there were also small municipalities with populations of only a few thousand in rural areas, and the Kanagawa Prefecture had a very diverse socioeconomic background municipalities. There was a variation in SES by population size (S8 Table). From these points of view, the findings of this study could be applied to regions with various social backgrounds. As noted above, there were issues regarding the definition of SES, but this was also indicated by the fact that similar trends were observed in this study as in other countries with different insurance systems [15].

-9) Page18: L366-367

Regarding age adjustment, at least for mortality, age-adjusted mortality rate for each cancer type can be calculated by applying for secondary use of Vital Statistics. If the authors cannot calculate age-adjusted cancer incidence and mortality from the data provided Kanagawa cancer registries, a more accurate verification is possible with alternative methods. Please consider the additional analysis or the future study.

Reply:

Thank you for your suggestion. We additionally performed Poisson models using age-adjusted mortality as response variables and demonstrated no specific trend among community SES and cancer mortality (S11 Table). We considered this is mainly because Vital Statistics included cancer-death people without pathological diagnosed, causing misclassification. For this reason, we have added the following sentence and S11,S12 Table.

Line 217: As for mortality, age-adjusted rates were available from Vital Statistics, and we additionally performed a Poisson regression analysis using age-adjusted mortality rate of each cancer as the response variable. Vital Statistics compiles data from the family register including deaths from all municipalities in Japan. Vital Statistics contains data on date of decease, cause of death, and address.

Line 276: The results of the correlation coefficient, VIF of the Poisson regression, multilevel analysis, t-test by population size, Linear regression using quartiles of community SES, morbidity and mortality for each year, the number of deaths for each type of cancer registered in the vital statistics, and the correlation coefficient using age-adjusted mortality as response variables are shown in S1–S12 Tables.

Line 509: Using age-adjusted mortality as response variables demonstrated no specific trend among community SES and cancer mortality (S12 Table). We considered this because Vital Statistics included cancer-death individuals without pathological diagnosis and DCO data, meaning non-exact cancer data, causing misclassification. Comparing Table 1 and S11 Table, there was a discrepancy in the number of cancer deaths between cancer registry data and vital statistics, and this discrepancy became larger the further back in time the data went. Another possibility is that residual confounding, which could not be adjusted by including the aging rate as an explanatory variable, may have been eliminated through age adjustment.

Minor points

-1. Methods: Cancer registry data

How many municipalities were included in this analysis? Please add the explanation of geographical unit in Methods section.

Reply:

Thank you for your suggestion. We have added the following sentence:

Line 141: In total, 58 municipalities were included in the analysis.

-2. Methods: Cancer registry data

How did the authors deal with the changes in the composition of municipalities with regard to the mergers and consolidations that occurred between 2000-2016?

Reply:

Thank you for your question. We have added the following sentence:

Line 140: Municipalities in existence as of 2016 were included in the analysis; for new municipalities created between 2000 and 2016, pre-creation data were treated as NA.

-3. Methods: SES and other data (Page6: L 159-160)

The authors said that data on neighborhood income was fitted to the annual categories, but does it have to consider the changes in community-level SES between 2000-2016?

Reply:

Thank you for your question. This sentence was intended to say that since income data was only available every five years starting in 2003, the analysis was conducted by matching income data and cancer data at three-year intervals, for example, matching income data in 2003 with cancer data in 2000. We have revised this sentence as follow in S1 File:

Line 176: ~~Data on neighborhood income was fitted to the annual categories used for cancer morbidity and mortality.~~ As income data was only available every five years starting in 2003, the analysis was conducted by matching income data and cancer data at three-year intervals, for example, matching income data in 2003 with cancer data in 2000.

-4. Methods: SES and other data (Page6: L162-163)

It is not correct to cite Citation No. 25 here because this citation is regarding to land price.

Reply:

Thank you for your comment. We have revised the text as follows is S1 File:

Line 188: The employment rate is defined as the number of people in jobs in a municipality~~[25]~~[28] divided by the population of that municipality.

-5. Methods: SES and other data (Page6: L162-163)

Why the authors used the total population as the denominator for the calculation of the employment rate? Official statistics such as Population Census use population aged 15 and over as the denominator. Please explain the reason that the author used a different calculation method to that reported by national government.

Reply:

Thank you for your question. We modified the denominator of the employment rate to the labor force (as described in S1 below) and revised Table1-3, Fig 1, S1-4 Fig, S1-7 Table, S9 Table, and the following sentence:

Line 243: ~~In the model using the land price as the explanatory variable, the largest inverse regression coefficient of community SES indicator was -0.06 (95% confidence interval (CI) -0.10, -0.01) found in a model with the mortality of colorectal cancer as the response variable in men. In the model using the neighborhood income as the explanatory variable, the largest inverse regression coefficient of community SES indicator was -0.39 (95% CI -0.51, -0.27) found in a model with the mortality of liver cancer as the response variable in women. In the model using the education level as the explanatory variable, the largest inverse regression coefficient of community SES indicator was -0.12 (95% CI -0.24, -0.01) found in a model with the morbidity of liver cancer as the response variable in women. In the model using the employment rate as the explanatory variable, the largest inverse regression coefficient of community SES indicator was -0.49 (95% CI -0.65, -0.32) found in a model with the mortality of liver cancer as the response variable in men.~~ For morbidity, SES with a significant negative association with cancer in men were land price with lung and stomach cancer, neighborhood income with lung, stomach, and liver cancer, education level with lung cancer, and employment rate with lung, stomach, colorectal, and liver cancer. In women, associations found between cancers and SES were between land price with breast cancer, neighborhood income with stomach, colorectal, and liver cancer, education level with stomach and liver cancer, and employment rate with lung, colorectal, liver, and breast cancer. The largest inverse regression coefficients of community SES indicators were -0.15 (95% confidence interval (CI) -0.20, -0.10), found in the association of employment rate with liver cancer in men, and -0.39 (95% CI -0.53, -0.24), found in the association of employment rate with liver cancer in women. For mortality, SES with a significantly negative association with cancer in men were land price with colorectal cancer, neighborhood income with lung, stomach, colorectal, and liver cancer, education level with lung and colorectal cancer, and employment rate with lung, stomach, colorectal, and liver cancer. In women SES with a significantly negative association with cancer were neighborhood income with colorectal and liver cancer, education level with stomach and liver cancer, and employment rate with all cancer types. The largest inverse regression coefficients of community SES indicators were -0.28 (95% CI -0.35, -0.22), found in the association of employment rate with liver cancer in men, and -0.91 (95% CI -1.11, -0.70), found in the association of employment rate with liver cancer in women.

Line 342: This study revealed that ~~the community neighborhood income and employment rate had significant inverse associations with cancer morbidity and mortality rates in 13 and 15 of the 18 Poisson regression models, respectively.~~ for morbidity, neighborhood income exhibited a significant negative association with lung, stomach, and liver cancer in men and stomach, colorectal, and liver cancer in women, and employment rate with lung, stomach, colorectal, and liver cancer in men and lung, colorectal, liver, and breast cancer in women; for mortality, neighborhood income had a significant negative association with lung, stomach colorectal, and liver cancer in men and colorectal and liver cancer in women, and employment rate with lung, stomach, colorectal, and liver cancer in men and all cancer types in women.

Line 376: Cancer mortality rates did not have specific association with neighborhood income and employment rate in previous study [16]; this result differs from our results, passively owing to the definition of the neighborhood income and ~~employment rate~~ the methods of obtaining data on cancer patients. Further studies are needed to investigate the indicators of neighborhood income ~~and employment rate~~ most associated with cancer and on methods of obtaining data on patients with cancer.

Line 383: Even though the community SES indicators changed by one standard deviation, the change in morbidity and mortality averaged approximately ~~0.9~~0.1 per 100 000 people/year.

Line 400: Among the four community SES indicators, the employment rate had the highest mean absolute value of the regression coefficient: land price, 0.08; neighborhood income, 0.08; education level, 0.05; and employment rate, ~~0.17~~0.21.

-6. Methods: SES and other data (Page6: L162-163)

Please provide the rationale as a high school education, not a university graduate.

Reply:

Thank you for your suggestion. We have added the following sentence in S1 File:

Line 182: ~~In some areas of Kanagawa Prefecture during the period covered by the data used in this study, the percentage of college graduates in the population was low at about 15%, and it was possible that those who went on to college alone would not cover the high educational level of the population at that time. Therefore, in this study, we defined higher education level as those who were educated in higher educational institutions than high school.~~

- 7. Result

Although the indicator with the highest inverse association were listed, it would be more appropriate to list those with a strong impact on incidence and mortality, respectively in order to examine preventive strategy for incidence and mortality separately.

Reply:

Thank you for your suggestion.

We made revisions with this suggestion when responding to the points raised by “Reviewer#1 Minor points 5”.

- 8. Discussion: Page15: L256-258

It would be more appropriate to list the cancer types that were found to be associated with income and employment rate rather than the model number. In addition, the current description makes the correspondence between the model number and the results unclear. Please provide more detailed description about summary results.

Reply:

Thank you for your suggestion.

We made revisions with this suggestion when responding to the points raised by “Reviewer#1 Minor points 5”.

-9. Discussion: Page16: L311- L312

The previous study cited were from Canada and USA, and there may be difference in the insurance system. It would be important to consider the differences between Japan, which has a universal health insurance system, and the USA, which does not.

Reply:

Thank you for your suggestion. We have revised the following sentence:

Line 398: It is possible that these unique insurance systems in Japan, which differ from those in other countries, had an impact on the results of our study.

-10. Discussion: Page16: L311- L312

In Japan, there is a study that have examined the association between cause-specific mortality including cancer type and final education. In this previous study, it has already been shown that mortality rate by cancer type varied by final education. There is a difference between individual level in this previous study and community level in the present study, and the authors pointed out, there is a possibility that the results may not be properly evaluated by using the final education as educational level. However, I think that this assertion would be contrary to the results already presented in Japan. I hope the authors will reconsider this after reviewing the previous study I have suggested.

H Tanaka, et al. Educational inequalities in all-cause and cause-specific mortality in Japan: national census-linked mortality data for 2010-2015. Int J Epidemiol; 53 (2): dyae031.

Reply:

Thank you for your suggestion. As mentioned, cancer and final education are considered to be associated at the individual level in Japan, so this paper has been revised to the following description, which focuses on the use of final education in the assessment of educational level at the regional level:

Line 408: Community land prices and educational levels did not show specific associations with cancer. Regarding land prices, this study is the first statistical analysis of the associations between land price and the morbidity or mortality of several cancer types, but land price differences existed within the same municipality. Further detailed information on addresses is required to treat the land prices as community SES indicators because our cancer registry data did not contain such detailed information. Community education level is considered an important community SES indicator because it influences the early stages of life and plays a role in personality development [21]. However, the average years of education and final education rate were substituted for education levels because community education levels are difficult to assess, suggesting that the community education level was not accurately assessed [21]. This may have affected the association between community education levels and cancer in this study (Table 2,3). Previous research defined community education levels as the number of university graduates per 1 000 people and demonstrated that communities with high educational levels continued to have higher cancer mortality [16], unlike that noted in our study results. Many studies have used years of education or final education as factors of community SES to demonstrate the association between community SES and cancer [16,18,19]. However, community education level should be used with caution in community SES and cancer research, given the aforementioned challenges. Regarding the association between community education level and morbidity rate of cancer, this study is the first statistical analysis. Significantly positive associations were noted between cancer incidence and mortality for land price and education level. Notably, for colorectal cancer in women, both land price and education level were associated with increased morbidity and mortality. Therefore, implementing targeted colorectal cancer control for women living in areas with higher land prices or education levels may be effective. It is important to consider such individualized cancer control measures in the community SES. One possibility is that the interaction between individual SES and community SES may have increased the risk of further cancer in individuals with low individual SES living in a high community SES due to the interaction. However, because this study was an ecological study and did not measure individual SES, the above possibility could not be verified. Therefore, it is important to verify cancer control measures that focus on various community SES levels.

-11. Discussion: Page16: L314- L317

From which results did this description refer? Please indicate the relevant table in parentheses.

Reply:

Thank you for your comment. We have added the relevant table in parentheses as follows:

Line 418: This may have affected the association between community education levels and cancer in this study (Table 2,3).

-12. Discussion: Page17: L349- L350

The authors showed the actual status of cancer incidence and mortality within Kanagawa prefecture. It is necessary to examine and discuss based on the cancer screening uptake rate by municipality not the prefectural level screening uptake rate. I suggest to examine the community-level SES and cancer screening uptake rate using official statistics such as Report on Regional Public Health Services and Health Promotion Services or Annual report by Kanagawa cancer registry.

Reply:

Thank you for your suggestion. We have conducted additional analysis (S1 Table) and following sentence:

Line 532: Furthermore, we examined the correlation between community SES and published data on health checkup uptake rates by municipality in the Kanagawa Prefecture [42] (S1 Table). No clear correlation was found, possibly because the health checkup uptake rate data used in this analysis only counted the number of examinations conducted by the municipality, and did not count those conducted by companies.

Line 711: 42. Kanagawa Prefecture screening uptake rate by city, town, and village. [Cited 2024 September 16]. Available from: https://www.e-stat.go.jp/stat-search?page=1&toukei=00450025&bunya_l=15

Reviewer #2:

Background

-1. Lines 101-102: Consider putting information on the source of data in the Methods section.s

Reply:

Thank you for your comment.

We have revised it as described in response to the Major points, Materials and Methods, 1) from Reviewer #1.

Methods

-2. Lines 125-126: Please explain why data from the registry could not be analyzed using the person-year method.

Reply:

Thank you for your comment. We have revised the following sentence:

Line 131: The Kanagawa Cancer Registry is created by medical institutions registering data when a cancer diagnosis or death occurs. This registry however does not contain data on pre-morbid observations from individuals. Therefore, ~~Because~~ data from the Kanagawa Cancer Registry could not be analyzed using the person-year method, and we calculated the morbidity and mortality (per 100 000 population/year) every 5 years (2000, 2005, 2010, and 2015) in each community and sex after obtaining population data from the Portal Site of Official Statistics of Japan [26].

-3. Line 128-129: Are Yokohama, Kawasaki and Sagamihara municipalities? This could be made clearer, e.g. “Each municipality was defined as a community; the municipalities of Yokohama, Kawasaki and Sagamihara were divided into wards.”

Reply:

Thank you for your comment. We have revised as you suggested.

Line 138: Each municipality was defined as a community; the municipalities of Yokohama, Kawasaki, and Sagamihara were divided into wards.

-4. Lines 146 – 158: This calculation example could go in the supplementary material. Please consider having a supplementary table showing all four measures of SES, the source of data of each and how the measures were calculated.

Reply:

Thank you for your recommendation. We have created another supporting information file (S1 File) and moved the community SES information there as follows.

Line 150: The definition of SES, calculation method, source, and value of each data are described in S1 File. ~~We obtained income, education level, and employment data in each municipality from the Portal Site of Official Statistics of Japan[24]. We also obtained land price data from the Kanagawa prefectural Government website[25]. We also obtained land price data from the Kanagawa prefectural Government website. The obtained income data were the number of families with incomes of less than 3 000 000, 3 000 000-5 000 000, 5 000 000-7 000 000, 7 000 000-10 000 000, 10 000 000-15 000 000, and more than 15 000 000 Japanese Yen per community. We defined each income as 1 500 000, 4 000 000, 6 000 000, 8 500 000, 12 500 000, and 20 000 000 Japanese Yen, respectively, and calculated the average income by multiplying each income and number of families per income, summing them up, and finally dividing by the number of all families in each year and community. We defined those average incomes as the neighborhood income. For example, the average income in a given region and year was calculated as follows:~~

$$\text{Neighborhood income}$$

$$= (1 500 000 \times number of families with income less than 3 000 000 Japanese Yen$$

$$+ 4 000 000 \times number of families with family income form 3 000 000 to 5 000 000$$

$$+ 6 000 000 \times number of families with family income form 5 000 000 to 7 000 000$$

$$+ 8 500 000 \times number of families with family income form 7 000 000 to 10 000 000$$

$$+ 12 500 000 \times number of families with family income form 10 000 000 to 15 000 000$$

$$+ 20 000 000 \times number of families with income more than 15 000 000)$$

$$/ the number of all families$$

~~Data on neighborhood income was fitted to the annual categories used for cancer morbidity and mortality. Since income data was only available every five years starting in 2003, the analysis was conducted by matching income data and cancer data at three-year intervals, for example, matching income data in 2003 with cancer data in 2000. We converted the land price and neighborhood income to U.S. dollars (1$ = 133 Japanese Yen, the rate on March 20~~^~~th~~^~~, 2023). We defined the community education level as the proportion of people with more than a high school education in each community. In some areas of Kanagawa Prefecture during the period covered by the data used in this study, the percentage of college graduates in the population was low at about 15%, and it was possible that those who went on to college alone would not cover the high educational level of the population at that time. Therefore, in this study, we defined higher education level as those who were educated in higher educational institutions than high school. The employment rate is defined as the number of people in jobs in a municipality[25] divided by the population of that municipality. In community SES data, only employment rates can use gender-disaggregated data.~~

-5. More descriptive information on the municipalities would be beneficial to understand the Kanagawa prefecture better. For example, how many municipalities are there in the Kanagawa prefecture? How many are urban / town / rural and what is the population range of each type of municipality?

Reply:

Thank you for your suggestion.

The total number of municipalities in Kanagawa was added as Reviewer#1 Minor points -1 suggestion. The population range of each type of municipality was added in S9 Table. As for the number of municipalities in urban, town, and rural areas were added as follows:

Line 212: The number of municipalities in urban, town, and rural areas were 3 (28 yard), 16, and 14, respectively.

-6. Please make it clear how you formatted / prepared your data for the analysis - e.g. at what level the data were analysed.

Reply:

Thank you for your comment. We have added that information in S1 file and the following sentence:

Line 149: We also formatted community SES data by each municipality, every 5 years, and by sex, if available, the same as cancer morbidity and mortality formatting.

-7. Lines 174-175, please explain why “other models adding the municipality code as the explanatory variable were assessed”.

Reply:

Thank you for your comment. We have added the following sentence:

Line 201:Additionally, other models that incorporated adding the municipality code as an explanatory variable to account for features unique to the region beyond SES were assessed.

-8. Line 177: Please explain why the multilevel analysis was performed.

Reply:

Thank you for your comment. We have added the following sentence:

Line 206: Furthermore, we performed the multilevel analysis by year and municipality code to examine the fixed effect and random effect by year and municipality using the R “lmer” function in the “lme4” package.

Results

-9. Lines 190-192: Please consider reporting incidence and mortality as relative values (i.e. relative to the overall population) rather than reporting total numbers.

Reply:

Thank you for your comment. We have revised the following sentence:

Line 233: The average incidence per 100 000 population of lung, stomach, colorectal, liver, and breast cancers was 36.1, 45.5, 64.4, 6.5, and 75.0 and the number of average corresponding deaths per 100 000 population was 22.4, 15.3, 15.0, 3.6, and 10.3, respectively. ~~The total incidence of lung, stomach, colorectal, liver, and breast cancers was 59 021, 74 241, 118 825, 10 807, and 60 894 and the number of corresponding deaths was 10 524, 10 366, 13 027, 1 892, and 3 419, respectively.~~

-10. The above comment (9) also applies to Table 1, please consider reporting relative values as well as total numbers.

Reply:

Thank you for your comment. We have revised Table 1.

-11. Table 1: “Death due to cancer” – please clarify in the Methods section where you obtained information on cause of death from, to derive the cancer-related deaths reported in this table.

Reply:

Thank you for your comment. We have added the following sentence:

Line 130: Information on diagnosis and death, such as date, was obtained from cancer registries.

-12. General comment: this section of the manuscript is too brief and doesn’t go into much detail of explaining the results of Tables 2 and 3. It only summarises the largest inverse regression coefficient of each SES indicator. I would suggest reframing how you write this section. For example, firstly report on the results of morbidity: which of the SES indicators were inversely associated with morbidity and for which cancers? Give some examples of results. Then report on the results for mortality in a similar way.

Reply:

Thank you for your comment.

We made revisions with your suggestion when responding to the points raised by “Reviewer#1 Minor points 7”.

Discussion

-13. Consider discussing your findings in the context of different types of municipalities (Table S8 wasn’t referred to in the manuscript?).

Reply:

Thank you for your comment.

We made revisions with your suggestion when responding to the points raised by “Reviewer#1 Major points Results 1)”. Please note that due to the addition of a table, the original Table 8 is now Table 9.
