# Supplementary material for: Association between socioeconomic background and cancer: An ecological study using cancer registry and various community socioeconomic status indicators in Kanagawa, Japan
Source: PLoS One. 2025 Jul 9;20(7):e0326895. doi: 10.1371/journal.pone.0326895 (PMC12240336; doi:10.1371/journal.pone.0326895)
Supplement: Attachment — Submitted filename: Response_to_Reviewers.docx [file pone.0326895.s004.docx]

**Journal Requirements:
-Please review your reference list to ensure that it is complete and correct. If you have cited papers that have been retracted, please include the rationale for doing so in the manuscript text, or remove these references and replace them with relevant current references. Any changes to the reference list should be mentioned in the rebuttal letter that accompanies your revised manuscript. If you need to cite a retracted article, indicate the article’s retracted status in the References list and also include a citation and full reference for the retraction notice.**

Reply:

Thank you for your comment. Accordingly, we have removed the retracted reference, replaced it with another current reference, and have revised the corresponding text in the manuscript as follows:

Line 62: ~~Disparities in SES are widening in many countries~~ Regional disparities in SES within each country remain a challenge in many countries [14].

Line 569: 14. ~~Socioeconomic status differences within each country~~ Inequalities in regional economic performance within countries. [Cited 2025 March 23]. Available from: [~~https://www.oecd-ilibrary.org/docserver/reg_glance-2016-en.pdf?expires=1663625072&id=id&accname=guest&checksum=3C151B90E6109390EBCD27C86FF6E9E1~~](https://www.oecd-ilibrary.org/docserver/reg_glance-2016-en.pdf?expires=1663625072&id=id&accname=guest&checksum=3C151B90E6109390EBCD27C86FF6E9E1) https://www.oecd.org/en/publications/oecd-regional-outlook-2019_9789264312838-en.html.

**Reviewers' comments:**

**Reviewer #1
-1. Result (Line 277-281)
The explanations of results have appeared in Discussion section, but should be better included in Result section.
I think the current description would be the explanation of S1-S12 and does not describe the results shown from the studies carried out.**

Reply:

Thank you for your comment. Accordingly, we have revised the text as follows:

Line 189: ~~Figure 1 and S1–S4 Figs present scatterplots of the association between community SES with each cancer morbidity and mortality for men and women in Kanagawa, Japan, 2000–2015. Tables 2 and 3 show the regression coefficient of each community SES indicator by Poisson regression using community SES, aging rate, and year as explanatory variables.~~

Line 211: With a change of one standard deviation in the community SES indicators, the change in morbidity and mortality averaged approximately 0.1 per 100 000 people/year. The mean absolute values of the regression coefficients in land price, neighborhood income, education level, and employment rate were 0.08, 0.08, 0.05, and 0.21, respectively (Figure 1, S1–S4, Table 2, 3).

Line 216: ~~The results of the correlation coefficient, VIF of the Poisson regression, multilevel analysis, t-test by population size, Linear regression using quartiles of community SES, morbidity and mortality for each year, the number of deaths for each type of cancer registered in the vital statistics, and the correlation coefficient using age-adjusted mortality as response variables are shown in S1–S12 Tables.~~ The aging rate and employment correlation coefficients were -0.65 (95% CI -0.75, -0.52) in men and -0.29 (95% CI -0.47, -0.10) in women (S1 Table). All VIF values were less than 2.0 in the models using community SES indicator, aging rate, and year as explanatory variables (S2 Table). Some VIF values were over 5 in the models using community SES indicator, aging rate, year, and municipality code as explanatory variables (S3 Table). The maximum random effect variance in multilevel analyses classified by year was 84.1 (S4, S5 Tables) and that classified by municipality code was 98.0 (S6, S7 Tables). Multilevel analyses classified by municipality code in the education-level model could not be performed (S6, S7 Tables). The average correlation coefficients by year of land price, neighborhood income, education level, employment rate in men, and employment rate in women were 0.98, 0.86, 0.97, 0.73, and 0.92, respectively (S8 Table). Significant differences (those for which P value < 0.05) were observed in mortality for liver and breast cancer between the town and the rural areas, and in morbidity for stomach cancer and in mortality for stomach, liver, and breast cancers between the urban and the rural areas (S9 Table). In linear regressions using quadratized community SES as explanatory variable and morbidity or mortality rate in each year as response variable, SES with significantly lower morbidity rates in the fourth quartile than in the first quartile were stomach cancer in men and stomach, liver, and breast cancer in women for land price, lung, stomach, colorectal, and liver cancer in men and stomach, colorectal, and liver cancer in women for neighborhood income, lung, and liver cancer in men and liver cancer in women for education level, and lung, colorectal, liver cancer in men and colorectal cancer in women for employment rate and mortality are lung, stomach colorectal cancer in men for land price, lung, stomach, colorectal, and liver cancer in men and stomach colorectal, and liver cancer in women for neighborhood income, lung, colorectal, and liver cancer in men for education level, and stomach, colorectal, and liver cancer in men and liver and breast cancer in women for employment rate (S10 Table). The number of average corresponding deaths owing to lung, stomach, colorectal, liver, and breast cancers registered in the vital statistics per 100 000 population was 41.6, 32.9, 30.9, 20.1, and 18.6, respectively (S11 Table). Regarding regression coefficients of community SES indicators in a Poisson regression model using community SES indicator and year as explanatory variables and age-adjusted mortality from Vital Statistics as response variable, in men, there was significant negative association of land price with stomach and liver cancer; of neighborhood income with lung, stomach and liver cancer; of education level with lung, stomach, and liver cancer; and of employment rate with lung, stomach, and liver cancer. In women, there was significant negative association of land price with stomach, liver, and breast cancer; of neighborhood income with liver cancer; and of education level with stomach and liver cancer (S12 Table).

Line 303: This study ~~revealed that for morbidity, neighborhood income exhibited a significant negative association with lung, stomach, and liver cancer in men and stomach, colorectal, and liver cancer in women, and employment rate with lung, stomach, colorectal, and liver cancer in men and lung, colorectal, liver, and breast cancer in women; for mortality, neighborhood income had a significant negative association with lung, stomach colorectal, and liver cancer in men and colorectal and liver cancer in women, and employment rate with lung, stomach, colorectal, and liver cancer in men and all cancer types in women. This~~ suggests that the community neighborhood income or employment rate was associated with considerable incidences of cancer and risks of death.

Line 338: ~~Even though the community SES indicators changed by one standard deviation, the change in morbidity and mortality averaged approximately 0.1 per 100 000 people/year.~~

Line 354: Among the four community SES indicators, the employment rate had the highest mean absolute value of the regression coefficient~~: land price, 0.08; neighborhood income, 0.08; education level, 0.05; and employment rate, 0.21~~.

Line 421: Areas with low community employment rates may have high cancer morbidity or mortality rates because of the high aging rate in men~~, according to the results of the correlation coefficients between employment and aging rates~~ (S1 Table).

Line 426: However, ~~the VIF of the aging and employment rates was less than 2.0 in the models using community SES indicator, aging rate, and year as explanatory variables; thus~~ from the VIF results (S2 Table), we determined that no multicollinearity affected the results ~~(S2 Table)~~.

Line 429: On the contrary, the VIF in the models using community SES indicator, aging rate, year, and municipality code as explanatory variables~~, the VIF of several models were over 5, indicating~~ indicated the probability of multicollinearity, and these models were deemed inappropriate (S3 Table).

**-2. S8_Table
In S8_Table, is it the results of correlation between aging rate and SES indicator? If you examined the correlation with year, please include the results in this table.**

Reply:

Thank you for your comment. In this text, we intended to discuss SES changes over time; accordingly, we have revised S8 Table and the following sentences:

Line 161: Other analyses of correlation coefficients ~~between~~ of community SES ~~and~~ by year were conducted to confirm changes in SES over time.

Line 229: The average correlation coefficients by year of land price, neighborhood income, education level, employment rate in men, and employment rate in women were 0.98, 0.86, 0.97, 0.73, and 0.92, respectively (S8 Table).

Line 686: **S8 Table.** Correlation coefficients of ~~year and~~ community SES indicators by year in Kanagawa, Japan, 2000–2015 (DOCX).

**Reviewer #2
-Firstly, the authors have addressed my previous comments, but I would suggest further edits to the Results section to help the reader follow and digest the results being described. For example, including (in brackets) which Table(s) or Figures(s) the results they are referring to come from.**

Reply:

Thank you for your comment. We revised the text in response to your comment, as described in our reply to “Reviewer#1 1”, and as follows:

Line 182: ~~Table 1 summarizes the population, aging rate, community SES indicators, cancer incidence, and death due to cancer.~~ Between 2000 and 2015, the population of Kanagawa prefecture increased from approximately 8.5 to 9.3 million, and the aging rates increased from 12.9% to 23.3% and 16.4% to 27.7% for men and women, respectively. The average incidence per 100 000 population of lung, stomach, colorectal, liver, and breast cancers was 36.1, 45.5, 64.4, 6.5, and 75.0, respectively, and the number of average corresponding deaths per 100 000 population was 22.4, 15.3, 15.0, 3.6, and 10.3, respectively (Table 1).

Line 189: ~~Figure 1 and S1–S4 Figs present scatterplots of the association between community SES with each cancer morbidity and mortality for men and women in Kanagawa, Japan, 2000–2015. Tables 2 and 3 show the regression coefficient of each community SES indicator by Poisson regression using community SES, aging rate, and year as explanatory variables.~~

**-Secondly, I noticed some language errors (e.g. line 48-49: "Cancer is associated with high morbidity and mortality rates, making its prevention is important"), and suggest a further proofread.**

Reply:

Thank you for your comment. We have revised the text as follows:

Line 43: ~~Cancer is associated with high morbidity and mortality rates~~ Cancer has high morbidity and mortality rates, making its prevention ~~is~~ important.

Line 46: Low ~~income~~ SES is associated with high morbidity in individuals with lung cancer [1] and this may be because of the high smoking rates among individuals with low ~~income~~ SES [3].

Line 50: Some individual SES-focused cancer prevention studies have been introduced, including breast and cervical cancer screening and treatment provisions for low-income or non-insured women~~, who often have low participation rates in health rates~~; however, participation rates in these initiatives remain low [5].

Line 55: The reasons for this were not only economic barriers, but also ~~poor medical access and~~ regional socioeconomic background factors, such as poor medical access [8–11].

Line 59: They include the introduction of cancer screening considering regional ~~religion~~ culture and race [12], and community-based programs for Hepatitis B screening [13]. These interventions have enhanced participation rates and have been met with a high success rate ~~[13]~~.

Line 70: Few studies have investigated the association between each community SES indicator, such as neighborhood income or education level, and cancer as follows: the association between low community income with the morbidity of cervical, head and neck, lung, and gastrointestinal cancers, that of high community income with the morbidity of breast and prostate cancers [15], and that of the mortality of several cancer types with SES indicators [~~15,~~16].

Line 84: Another challenge ~~was~~ is that each community SES indicator ~~had~~ has its characteristics and ~~affected~~ affects people in different stages of life [21,22]. For example, the effects of the education level are observed from the early stage of life, while those of employment are observed after graduation ~~[21,22]~~.

Line 121: Data on cancer were classified by year, sex ~~(only women patients were included in breast cancer),~~ and community. For breast cancer, only women were included.

Line 155: ~~These models also included all community SES indicators while considering interaction.~~ Furthermore, a model that tested the interaction with all community SES indicators as explanatory variables was assessed.

Line 380: Significantly positive associations were noted between cancer incidence and mortality for land price and education level in some models.

Line 470: High community SES areas may have a high morbidity rate of cancer ~~morbidity~~ owing to the high uptake rate of cancer screening.

Line 498: Finally, we could not use age-stratified population data by municipality and year, and thus, we could not calculate the age-adjusted morbidity and mortality in the model using cancer registry data.
