# Supplementary material for: Association between socioeconomic background and cancer: An ecological study using cancer registry and various community socioeconomic status indicators in Kanagawa, Japan
Source: PLoS One. 2025 Jul 9;20(7):e0326895. doi: 10.1371/journal.pone.0326895 (PMC12240336; doi:10.1371/journal.pone.0326895)
Supplement: Attachment — Submitted filename: Response_to_Reviewers_auresp_3.docx [file pone.0326895.s005.docx]

Journal Requirements:

-Please review your reference list to ensure that it is complete and correct. If you have cited papers that have been retracted, please include the rationale for doing so in the manuscript text, or remove these references and replace them with relevant current references. Any changes to the reference list should be mentioned in the rebuttal letter that accompanies your revised manuscript. If you need to cite a retracted article, indicate the article’s retracted status in the References list and also include a citation and full reference for the retraction notice.

Reply:

Thank you for your comment. We have confirmed that none of the cited references have been retracted.

Reviewer #1:

-Many thanks for addressing my previous comments and I appreciate to opportunity for reviewing the revised manuscript. However, I concern the revised Result section has become complicated.

In my previous comments, I did not intend for authors to include all detailed results of supplementary materials in Result sections.

It is considered desirable that the most important results of supplementary materials should be described in Result section. However, I suggest that other results should be simplified, e.g. those results that can be presented together with the main results should be listed with only the table or figure number without detailed values, and those results that have been discussed for the Discussion should be listed with only the table or figure number after the description.

Reply:

Thank you for your comment. Accordingly, we have revised the text as follows:

(Page 9, Line 209–211)

~~The aging rate and employment correlation coefficients were -0.65 (95% CI -0.75, -0.52) in men and -0.29 (95% CI -0.47, -0.10) in women (S1 Table).~~ Correlation coefficients of the aging rate, screening rate, and community SES indicators are presented in S1 Table.

(Page 9, Line 218– Page 10, Line 221)

~~The average correlation coefficients by year of land price, neighborhood income, education level, employment rate in men, and employment rate in women were 0.98, 0.86, 0.97, 0.73, and 0.92, respectively (S8 Table).~~ Correlation coefficients of community SES indicators by year are presented in S8 Table.

(Page 10, Line 221–226)

~~Significant differences (those for which P value < 0.05) were observed in mortality for liver and breast cancer between the town and the rural areas, and in morbidity for stomach cancer and in mortality for stomach, liver, and breast cancers between the urban and the rural areas (S9 Table).~~ The average and SD of land price, neighborhood income, education level, employment rate, morbidity, and mortality for each cancer type in urban, town, and rural areas are summarized in S9 Table.

(Page 10, Line 226–239)

~~In linear regressions using quadratized community SES as explanatory variable and morbidity or mortality rate in each year as response variable, SES with significantly lower morbidity rates in the fourth quartile than in the first quartile were stomach cancer in men and stomach, liver, and breast cancer in women for land price, lung, stomach, colorectal, and liver cancer in men and stomach, colorectal, and liver cancer in women for neighborhood income, lung, and liver cancer in men and liver cancer in women for education level, and lung, colorectal, liver cancer in men and colorectal cancer in women for employment rate and mortality are lung, stomach colorectal cancer in men for land price, lung, stomach, colorectal, and liver cancer in men and stomach colorectal, and liver cancer in women for neighborhood income, lung, colorectal, and liver cancer in men for education level, and stomach, colorectal, and liver cancer in men and liver and breast cancer in women for employment rate (S10 Table).~~ Results of linear regressions with SES quartiles, year, and aging rate as explanatory variables and cancer incidence or mortality as response variables are presented in S10 Table.

(Page 10, Line 239–243)

~~The number of average corresponding deaths owing to lung, stomach, colorectal, liver, and breast cancers registered in the vital statistics per 100 000 population was 41.6, 32.9, 30.9, 20.1, and 18.6, respectively (S11 Table).~~ The number of deaths for each type of cancer registered in the vital statistics are described in S11 Table.

(Page 10, Line 243–Page 11, Line 253)

~~Regarding regression coefficients of community SES indicators in a Poisson regression model using community SES indicator and year as explanatory variables and age-adjusted mortality from Vital Statistics as the response variable, in men, there was significant negative association of land price with stomach and liver cancer; of neighborhood income with lung, stomach and liver cancer; of education level with lung, stomach, and liver cancer; and of employment rate with lung, stomach, and liver cancer. In women, there was significant negative association of land price with stomach, liver, and breast cancer; of neighborhood income with liver cancer; and of education level with stomach and liver cancer (S12 Table).~~ Regression coefficients of community SES indicators by Poisson regression using community SES indicator and year as explanatory variables and age-adjusted mortality as the response variable are presented in S12 Table.

-In addition, duplicated the previous other reviewer's comments, I suggest that the description of results and Tables or Figures should be listed correspondingly, for example

L202　association of employment rate with liver cancer in women (Table 2).

L211　association of employment rate with liver cancer in women (Table 3).

Reply:

Thank you for your comment. Accordingly, we have revised the text as follows:

(Page 8, Line 178–180)

Between 2000 and 2015, the population of Kanagawa prefecture increased from approximately 8.5–9.3 million, and aging rates increased from 12.9% to 23.3% and from 16.4% to 27.7% for men and women, respectively (Table 1).

(Page 8, Line 184–187)

For morbidity, SES with a significant negative association with cancer in men were land price with lung and stomach cancer, neighborhood income with lung, stomach, and liver cancer, education level with lung cancer, and employment rate with lung, stomach, colorectal, and liver cancer (Figure 1, S1–S4 Figures, Table 2).

(Page 8, Line 187–191)

In women, associations found between cancers and SES were between land price with breast cancer, neighborhood income with stomach, colorectal, and liver cancer, education level with stomach and liver cancer, and employment rate with lung, colorectal, liver, and breast cancer (Figure 1, S1–S4 Figures, Table 2).

(Page 8, Line 191–Page 9, Line 194)

The largest inverse regression coefficients of community SES indicators were -0.15 (95% confidence interval (CI) -0.20, -0.10), found in the association of employment rate with liver cancer in men, and -0.39 (95% CI -0.53, -0.24), found in the association of employment rate with liver cancer in women (Table 2).

(Page 9, Line 194–198)

For mortality, SES with a significantly negative association with cancer in men were land price with colorectal cancer, neighborhood income with lung, stomach, colorectal, and liver cancer, education level with lung and colorectal cancer, and employment rate with lung, stomach, colorectal, and liver cancer (Figure 1, S1–S4 Figures, Table 3).

(Page 9, Line 198–201)

In women SES with a significantly negative association with cancer were neighborhood income with colorectal, liver, and breast cancer, education level with stomach cancer, and employment rate with all cancer types (Figure 1, S1–S4 Figures, Table 3).

(Page 9, Line 201–204)

The largest inverse regression coefficients of community SES indicators were -0.28 (95% CI -0.35, -0.22), found in the association of employment rate with liver cancer in men, and -0.91 (95% CI -1.11, -0.70), found in the association of employment rate with liver cancer in women (Table 3).

(Page 9, Line 204–206)

With a change of one standard deviation in the community SES indicators, the change in morbidity and mortality averaged approximately 0.1 per 100 000 people/year (Table 2, 3).

(Page 9, Line 206–208)

The mean absolute values of the regression coefficients in land price, neighborhood income, education level, and employment rate were 0.08, 0.08, 0.05, and 0.21, respectively ~~(Figure 1, S1–S4, Table 2, 3)~~(Tables 2–3).

Reviewer #2:

-Thank you for the opportunity to review this revised version of the manuscript. My previous comments have been addressed. I only have one minor comment remaining.

Lines 219-230 (text supporting S10 Table): for ease of reading, consider breaking this text into more than one sentence.

Reply:

Thank you for your comment. Considering Reviewer #1’s comment and as described in our response to Reviewer #1, this sentence has been revised to be more concise.
